# Development and validation of a lifestyle-based model for colorectal cancer risk prediction: the LiFeCRC score

**DOI:** 10.1186/s12916-020-01826-0

**Published:** 2021-01-04

**Authors:** Krasimira Aleksandrova, Robin Reichmann, Rudolf Kaaks, Mazda Jenab, H. Bas Bueno-de-Mesquita, Christina C. Dahm, Anne Kirstine Eriksen, Anne Tjønneland, Fanny Artaud, Marie-Christine Boutron-Ruault, Gianluca Severi, Anika Hüsing, Antonia Trichopoulou, Anna Karakatsani, Eleni Peppa, Salvatore Panico, Giovanna Masala, Sara Grioni, Carlotta Sacerdote, Rosario Tumino, Sjoerd G. Elias, Anne M. May, Kristin B. Borch, Torkjel M. Sandanger, Guri Skeie, Maria-Jose Sánchez, José María Huerta, Núria Sala, Aurelio Barricarte Gurrea, José Ramón Quirós, Pilar Amiano, Jonna Berntsson, Isabel Drake, Bethany van Guelpen, Sophia Harlid, Tim Key, Elisabete Weiderpass, Elom K. Aglago, Amanda J. Cross, Konstantinos K. Tsilidis, Elio Riboli, Marc J. Gunter

**Affiliations:** 1 Nutrition, Immunity and Metabolism Senior Scientist Group, Department of Nutrition and Gerontology, German Institute of Human Nutrition Potsdam-Rehbruecke (DIfE), Nuthetal, Germany; 2 Institute of Nutritional Science, University of Potsdam, Potsdam, Germany; 3 Department of Epidemiological Methods and Etiological Research, Leibniz Institute for Prevention Research and Epidemiology - BIPS, Bremen, Germany; 4 Division of Cancer Epidemiology, German Cancer Research Center (DKFZ), Heidelberg, Germany; 5 International Agency for Research on Cancer, World Health Organization, Lyon, France; 6 National Institute for Public Health and the Environment (RIVM), Bilthoven, The Netherlands; 7 Department of Epidemiology and Biostatistics, School of Public Health, Imperial College London, London, UK; 8 Department of Public Health, Aarhus University, Aarhus, Denmark; 9 Danish Cancer Society Research Center, Copenhagen, Denmark; 10 CESP, Faculté de Medicine, Université Paris-Saclay, Villejuif, France; 11 Institut Gustave Roussy, Villejuif, France; 12 Dipartimento di Statistica, Informatica e Applicazioni “G. Parenti” (DISIA), University of Florence, Florence, Italy; 13 Hellenic Health Foundation, Athens, Greece; 14 2nd Pulmonary Medicine Department, School of Medicine, National and Kapodistrian University of Athens, “ATTIKON” University Hospital, Haidari, Greece; 15 EPIC Centre of Naples, Dipartimento di Medicina Clinica e Chirurgia, University of Naples Federico II, Naples, Italy; 16 Cancer Risk Factors and Lifestyle Epidemiology Unit, Institute for Cancer Research, Prevention and Clinical Network – ISPRO, Florence, Italy; 17 Epidemiology and Prevention Unit, Fondazione IRCCS Istituto Nazionale dei Tumori di Milano, Milan, Italy; 18 Unit of Cancer Epidemiology, Città della Salute e della Scienza University-Hospital and Center for Cancer Prevention (CPO), Turin, Italy; 19 Cancer Registry and Histopathology Department, Provincial Health Authority (ASP), Ragusa, Italy; 20 Julius Center for Health Sciences and Primary Care, University Medical Center Utrecht, Utrecht University, Utrecht, The Netherlands; 21 Department of Community Medicine, Health Faculty, UiT-the Arctic university of Norway, Tromsø, Norway; 22 Escuela Andaluza de Salud Pública (EASP), Granada, Spain; 23 Instituto de Investigación Biosanitaria ibs. GRANADA, Granada, Spain; 24 Centro de Investigación Biomédica en Red de Epidemiología y Salud Pública (CIBERESP), Madrid, Spain; 25 Universidad de Granada, Granada, Spain; 26 Department of Epidemiology, Murcia Regional Health Council, IMIB-Arrixaca, Murcia, Spain; 27 Unit of Nutrition and Cancer, Cancer Epidemiology Research Program, Translational Research Laboratory, Catalan Institute of Oncology (ICO), Barcelona, Spain; 28 Bellvitge Biomedical Research Institute (IDIBELL), Barcelona, Spain; 29 Navarra Public Health Institute, Pamplona, Spain; 30 Navarra Institute for Health Research (IdiSNA), Pamplona, Spain; 31 Public Health Directorate, Asturias, Spain; 32 Ministry of Health of the Basque Government, Public Health Division of Gipuzkoa, Biodonostia Health Research Institute, Donostia-San Sebastian, Spain; 33 Department of Clinical Sciences, Division of Oncology and Pathology, Lund University, Lund, Sweden; 34 Department of Clinical Sciences in Malmö, Lund University, Lund, Sweden; 35 Department of Radiation Sciences, Oncology, Umeå University, Umeå, Sweden; 36 Wallenberg Centre for Molecular Medicine, Umeå University, Umeå, Sweden; 37 Cancer Epidemiology Unit, Nuffield Department of Population Health, University of Oxford, Oxford, UK; 38 Department of Hygiene and Epidemiology, University of Ioannina School of Medicine, Ioannina, Greece

**Keywords:** Colorectal cancer, Risk prediction, Lifestyle behaviour, Risk screening, Cancer prevention

## Abstract

**Background:**

Nutrition and lifestyle have been long established as risk factors for colorectal cancer (CRC). Modifiable lifestyle behaviours bear potential to minimize long-term CRC risk; however, translation of lifestyle information into individualized CRC risk assessment has not been implemented. Lifestyle-based risk models may aid the identification of high-risk individuals, guide referral to screening and motivate behaviour change. We therefore developed and validated a lifestyle-based CRC risk prediction algorithm in an asymptomatic European population.

**Methods:**

The model was based on data from 255,482 participants in the European Prospective Investigation into Cancer and Nutrition (EPIC) study aged 19 to 70 years who were free of cancer at study baseline (1992–2000) and were followed up to 31 September 2010. The model was validated in a sample comprising 74,403 participants selected among five EPIC centres. Over a median follow-up time of 15 years, there were 3645 and 981 colorectal cancer cases in the derivation and validation samples, respectively. Variable selection algorithms in Cox proportional hazard regression and random survival forest (RSF) were used to identify the best predictors among plausible predictor variables. Measures of discrimination and calibration were calculated in derivation and validation samples. To facilitate model communication, a nomogram and a web-based application were developed.

**Results:**

The final selection model included age, waist circumference, height, smoking, alcohol consumption, physical activity, vegetables, dairy products, processed meat, and sugar and confectionary. The risk score demonstrated good discrimination overall and in sex-specific models. Harrell’s C-index was 0.710 in the derivation cohort and 0.714 in the validation cohort. The model was well calibrated and showed strong agreement between predicted and observed risk. Random survival forest analysis suggested high model robustness. Beyond age, lifestyle data led to improved model performance overall (continuous net reclassification improvement = 0.307 (95% CI 0.264–0.352)), and especially for young individuals below 45 years (continuous net reclassification improvement = 0.364 (95% CI 0.084–0.575)).

**Conclusions:**

LiFeCRC score based on age and lifestyle data accurately identifies individuals at risk for incident colorectal cancer in European populations and could contribute to improved prevention through motivating lifestyle change at an individual level.

## Background

Colorectal cancer accounted for over 1.8 million new cases or 10% of all new cases of cancer worldwide in 2018 [1]. Worryingly, the global burden of colorectal cancer is expected to rise by 60% reaching 2.2 million new cases and 1.1 million deaths in 2030, with European countries ranking highest in the global statistics of colorectal cancer incidence and mortality [2]. The projected increase in colorectal cancer burden necessitates improved assessment of primary prevention strategies [2, 3]. Targeted prevention in an asymptomatic population that addresses potentially modifiable factors has potential for reducing lifestyle-associated long-term risk of colorectal cancer and represents a cost-effective approach to reduce the cancer burden [4, 5].

Lifestyle behaviours such as smoking, alcohol consumption, and poor diet have long been recognized to be associated with a higher risk of colorectal cancer [6–15]. Updated evidence on nutrition and cancer risk further highlighted the importance of risk factors such as body fatness (i.e. abdominal adiposity), adult-attained height, physical activity, high intake of red and processed meat and low intakes of whole grains, dairy products and fish [15, 16]. Despite accumulation of evidence, translation of lifestyle information into individualized colorectal cancer risk assessment strategies has not been implemented so far. Risk stratification may aid the identification of high-risk individuals, guide referral to screening and motivate lifestyle modification [17]. Individualized risk estimates in primary care may essentially aid behaviour change and complement preventive approaches to shifting population distributions of risk factors [17].

A number of colorectal cancer risk prediction models have been published over the last decade [18–21]. Most published models have been predominantly developed using data from American and Asian populations [18, 19]. We have previously validated several models in European populations based on data from UK Biobank and the European Prospective Investigation into Cancer and Nutrition (EPIC) cohort  studies [20]; however, several gaps remain to be addressed. First, only a few previous models have been developed based on prospective cohort data with long enough follow-up time to account for the potentially long latency period of colorectal cancer development [18]. Second, important emerging predictors related to nutrition and lifestyle such as abdominal fatness have not been considered [22]. Third, most models focused only on model development and did not address the full continuum of model development, validation and communication recommended in recent methodological guidelines for research on risk prediction (i.e. TRIPOD, Transparent Reporting of a multivariable Prediction model for Individual Prognosis or Diagnosis) [19, 23]. Fourth, previous models were mostly developed using logistic regression and did not account for time-to-event. New approaches such as penalized regression methods (i.e. elastic net regression) and machine learning algorithms (i.e. random survival forest) might offer additional means for model improvement [24, 25]. Finally, model communication to the wider public was generally not addressed by previous studies and was restricted to providing a formula to calculate individual absolute risk of colorectal cancer [18]. Graphical nomograms and web-based applications could further aid in facilitating model communication [26].

In this context, we aimed to develop and validate a lifestyle-based risk prediction model for the prevention of colorectal cancer in a population-based European cohort. We further aimed to construct a simple and widely applicable user-friendly risk calculator offering an estimate of colorectal cancer risk based on individual’s personal data.

## Methods

### Study design and data source

The lifestyle-based prediction model for colorectal cancer risk (LiFeCRC score) was developed using data collected within EPIC, a multicentre prospective cohort study comprising 521,324 participants aged 17 to 98 years at study baseline (predominantly 35 to 70 years) recruited between 1992 and 2000 across 23 centres in 10 European countries [27]. Participants included blood donors, screening participants, health-conscious individuals and the general population. Written informed consent was obtained from all participants before joining the EPIC study. Approval for the EPIC study was obtained from the ethical review boards of the International Agency for Research on Cancer and from all local institutions through which subjects were recruited for the EPIC study, as previously reported [28].

### Case ascertainment

The primary outcome was incident colorectal cancer. Cancer cases were identified through population cancer registries in Denmark, Italy, the Netherlands, Spain, Sweden and the UK. In France, Germany and Greece, a combination of methods was used including health insurance records, cancer pathology registries and active follow-up of study participants. Follow-up began at the date of enrolment and ended at the date of diagnosis of colorectal cancer, death or last complete follow-up. The last update of endpoint information was done up to 31 September 2010. Colon and rectal cancers were defined according to the 10th Revision of the International Statistical Classification of Diseases, Injuries and Causes of Death (ICD-10), proximal colon tumours include tumours in the cecum, cecal appendix, ascending colon, hepatic flexure, transverse colon and splenic flexure (ICD-10 codes C18.0–18.5); distal colon tumours include those in the descending colon (ICD-10 code C18.6) and sigmoid colon (ICD-10 code C18.7); and rectal tumours are those occurring at the rectosigmoid junction (ICD-10 code C19) or in the rectum (ICD-10 code C20). Only the first primary neoplasm was included in the analysis; non-melanoma skin cancer was excluded.

### Study population

**Fig. 1:**
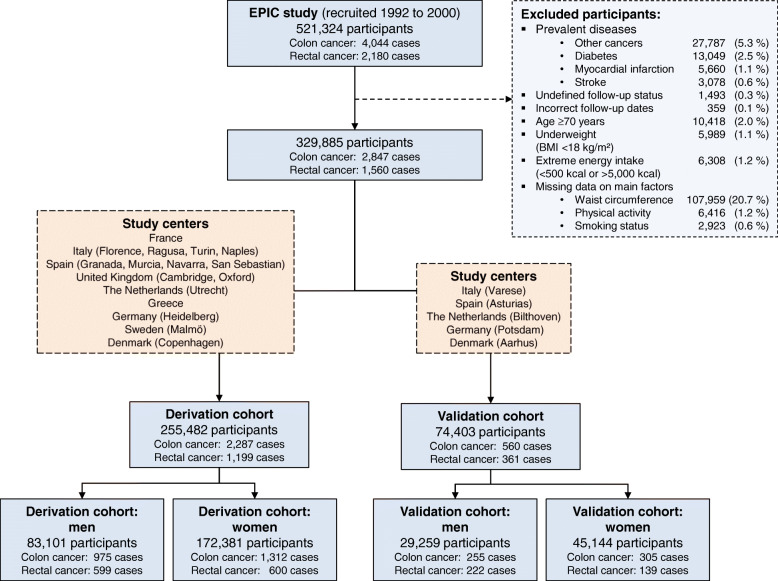
Flowchart of study population selection

Figure 1 presents a flowchart of study population selection for deriving the LiFeCRC score in the EPIC cohort. Participants with prevalent cancer, diabetes, myocardial infarction or stroke at recruitment and participants without follow-up were excluded. Missing information on main risk factors (sex, anthropometric measurements, lifestyle and dietary data) was present in 22.5% of the data, and therefore, entries with missing data were excluded for complete case analysis. Based on this, participants from EPIC-Umeå and EPIC-Norway were excluded from the current analyses due to lack of data on waist circumference measurements. The resulting study sample comprised 329,885 participants among which 4626 incident colorectal cancer cases (2847 colon cancer/1560 rectal cancer) were diagnosed during study follow-up. This sample was split into a derivation cohort (*N* = 255,482) and a validation cohort (*N* = 74,403) on a non-random principle following the TRIPOD recommendations [23]. The derivation sample included participants from 21 EPIC centres in France, Italy, Spain, UK, the Netherlands, Greece, Germany, Sweden and Denmark. The validation sample included participants representing Southern and Northern European populations from 5 EPIC centres in Italy, Spain, the Netherlands, Germany and Denmark (Fig. 1).

### Baseline data collection

At baseline, participants completed extensive medical, dietary and lifestyle questionnaires, including questions on alcohol use, smoking status, physical activity, education and previous illnesses. Body weight, height and waist circumference were measured in all centres except for EPIC-Oxford (health-conscious population) and EPIC-France where anthropometric measurements were self-reported [27]. Usual food intakes were measured by using country-specific validated dietary questionnaires [29]. All dietary variables used in the present study were calibrated by using an additive calibration method as previously described [30]. Non-steroidal anti-inflammatory drug (NSAID) use was only assessed in the Cambridge study center, and family history of colorectal cancer was assessed only in study centres in France, Spain and the UK. Baseline characteristics of participants with available information on NSAID use and family history of colorectal cancer are presented in Supplementary Table 1, Additional File 1.

### Model development

The model development and model validation were performed and reported following the TRIPOD guidelines [23, 31] (Supplementary Table 2, Additional File 1). The general workflow of model derivation, performance evaluation, validation and model communication are presented in Supplementary Fig. 1, Additional File 2.

Overall, the LiFeCRC score was derived based on beta coefficients for colorectal cancer risk estimated in Cox proportional hazard models within the derivation dataset. Time -to - event was defined as time from baseline assessment to first cancer event. Supplementary Table 3, Additional File 1 presents the variable names and measurement scales of a predefined set of 16 predictors selected based on published literature reflecting latest evidence from systematic reviews (i.e. World Cancer Research Fund/American Institute for Cancer Research reports) and based on availability of data in the EPIC cohort. Analyses based on Schoenfeld residuals and stratified Kaplan-Meier curves revealed no violation of the proportional hazard assumption of the Cox model. To test whether the predictive performance of each variable is the same, regardless of the values of other predictors, statistical interactions between different combinations of predictor variables on the multiplicative scale were tested using the likelihood ratio test. Since model discrimination was not improved by including significant interaction terms, the inclusion of interaction terms in the final Cox models was disregarded to avoid overfitting.

### Elastic net selection

Predictor variable selection was performed using bootstrapped elastic net regularization [32]. Elastic net regularization is a penalized regression method, combining least absolute shrinkage and selection operator (LASSO) and ridge regression. A penalty parameter *λ* is used to shrink predictor regression coefficients, eventually removing predictor variables from the model by setting their respective regression coefficient to zero. A mixing parameter *α* is used to fix the proportion for combining LASSO and ridge regression. Optimal values for both parameters *λ* and *α* were determined based on minimal mean error of 10-fold cross-validation using 100 possible *λ* values for *α* values between 0.5 and 1 (0.5, 0.6, 0.7, 0.8, 0.9, 1). The selected parameters were then used to bootstrap the elastic net regularization of each predictor’s Cox regression coefficient with 1000 replications. Based on all bootstrap replications, mean coefficient values and 95% confidence intervals were calculated for each predictor coefficient. Predictors with confidence intervals including zero were removed. All remaining predictors were then used to generate reduced elastic net penalized Cox regression models. The model selection was conducted for colorectal cancer as a single endpoint (LiFeCRC score) and according to sex and cancer subsite (colon/rectum). Variable selection and Cox regression modeling were performed using R 3.6.1 (R Core Team) [33], and the *glmnet* (version 2.0-18) [34] and *survival* (version 2.44-1.1) [35] packages.

### Absolute risk assessment

The individual 10-year absolute risk *P* (10y) for colorectal cancer was calculated using the following formula:

$$ P\left(10\mathrm{y}\right)=1-{S}_m{\left(10\mathrm{y}\right)}^{\exp \left({\mathrm{Risk}\ \mathrm{Score}}_i-{\mathrm{Risk}\ \mathrm{Score}}_m\right)} $$

The 10-year survival function estimate *S*_*m*_ (10y) was calculated for average predictor variable values. The average Risk Score_*m*_ and the individual Risk Score_*i*_ were computed using the following formulas:

$$ {\mathrm{Risk}\ \mathrm{Score}}_m=\sum \limits_j{\beta}_j\cdot \mathrm{predictor}\ {\mathrm{mean}\ \mathrm{value}}_j{\mathrm{Risk}\ \mathrm{Score}}_i=\sum \limits_j{\beta}_j\cdot {\mathrm{predictor}\ \mathrm{value}}_{ij} $$

The *j* index stands for a predictor variable of a Cox regression model and *β*_*j*_ is the beta estimate.

In additional analyses, the study population was stratified according to predefined risk categories of low, intermediate and high risk, based the 50th and 90th percentile of predicted risk in the derivation cohort. Incidence rates and model selection characteristics across the so defined risk categories in both the derivation and validation samples have been assessed.

### Model performance: discrimination and calibration

#### Model discrimination

Model discrimination was assessed based on Harrell’s C-index as a measure similar to the receiver operating characteristic statistic that takes the censored nature of data into account. This value represents the odds of the predicted probability of developing colorectal cancer being higher for those who actually develop colorectal cancer compared to those who do not develop the disease. To account for model optimism in terms of overfitting, bootstrapping with 1000 replications was performed. In bootstrapping, entries are randomly drawn with replacement from a data set until the bootstrap sample has the size as the original dataset. For each bootstrap sample, an elastic net penalized Cox regression model was fitted. Harrell’s C-index of each bootstrap model was then calculated for the bootstrap sample and the original data in each bootstrap replication. The difference of these values was averaged over all 1000 bootstrap replications to calculate the amount of optimism for the C-index of the original model, which was used to calculate an optimism-corrected C-index. This analysis was performed in R [33] with the package *rms* (version 5.1-3.1) [36].

#### Model calibration

Calibration plots of estimated individual predicted risks of developing colorectal cancer in the next 10 years were derived from the penalized Cox regression model. These values were divided into deciles, and each decile’s mean value was computed. The Kaplan-Meier survival function at 10 years with 95% confidence interval was calculated for each decile group. Subsequently, the trend of the mean predicted risks and the observed complement of the Kaplan-Meier survival of each decile was visually compared as a measure of calibration. Model performance, including Harrell’s C-index and calibration plots, was also evaluated in the validation cohort.

### Model communication

In order to assist the translation of the generated statistical model into an individual risk prediction equation, we created a 10-year risk assessment nomogram as a graphical model representation that allows risk estimation. For this purpose, we used the R [33] package *rms* (version 5.1-3.1) [36]. In addition, we developed a user-friendly risk calculator application using the R [33] packages *shiny* (version 1.2.0) [37] and *shinydashboard* (version 0.7.1) [38] that can be adapted for a web-based use. This application allows the prediction of individual colorectal cancer risk by including characteristics into input fields. The input values are then evaluated using the validated colorectal cancer risk prediction model.

### Random survival forest

Random survival forest was used as an alternative machine learning method in order to prove model robustness, i.e. assess whether the same set of predictors will be selected. Each random survival forest was generated with a total number of 500 decision trees with 100 unique data points on average in each terminal node and a maximum of 10 possible random split points to consider at each branch of a decision tree. A variable importance measure for each predictor variable, describing the impact of using randomly permuted values of this variable instead of observed values for the prediction of known entries, was then extracted from the random survival forest. For the computation of random survival forests, the package “randomForestSRC” (version 2.6.1) was used. Model performance was evaluated in the derivation and validation cohort using Harrell’s C-index and calibration plots.

### Sensitivity analyses

In sensitivity analyses, we evaluated the added predictive value of lifestyle data beyond age, using the following statistics: (1) improvement in model discrimination—based on goodness of fit (likelihood ratio test), estimated net change in Harrell’s C-index and continuous net reclassification improvement (NRI^> 0^); (2) improvement in model calibration based on comparison of calibration plots and (3) net benefit of the model based on decision curve analysis. We also stratified the study population in the derivation and validation sample according to age groups: < 45 years; 45–65 years; > 65 years and calculated model performance characteristics (Harrell’s C-index and NRI^> 0^) for the lifestyle-based model across these categories. In addition, we also calculated the predicted 10-year absolute risk of colorectal cancer for a predefined “healthy” and “unhealthy” lifestyle pattern across different age groups and a constant body height. In subsample of the derivation cohort with available information, Harrell’s C-index was compared between models with and without inclusion of NSAID use or family history. To address model generalizability, we further evaluated model performance across subgroups by selected variables, i.e. waist circumference, education, smoking status (including level of smoking intensity) and level of alcohol consumption. Finally, to account for the potential influence of competing risk of death (*N* = 23,774), we calculated the cumulative incidence adjusted for mortality and evaluated the discrimination of the reduced model based on Fine-Gray subdistribution hazard regression [39] in both the derivation and validation samples.

## Results

### Baseline characteristics

Table 1 shows the baseline characteristics of men and women in the derivation and validation cohorts. Overall, the distribution of risk factors was similar across both cohorts. In the derivation cohort, the mean age at study baseline was 51.4 years, 67.5% of the participants were women, and mean age at colorectal cancer diagnosis was 66.0 years in women and 66.4 years in men. Never-smokers, physically active and highly educated people comprised 49.1%, 10.3% and 24.6% of the derivation cohort, respectively. The median follow-up time was 15.4 (interquartile range 13.2 to 16.9) years in the derivation cohort and 14.1 (interquartile range 10.5 to 16.0) years in the validation cohort.

**Table 1:** Baseline characteristics of participants in the derivation and validation cohorts

Characteristics	Derivation cohort			Validation cohort		
	All participants	Men	Women	All participants	Men	Women
*N*	255,482	83,101	172,381	74,403	29,259	45,144
Age at recruitment, years, mean (SD)	51.4 (9.7)	52.3 (9.0)	51.0 (9.9)	49.7 (9.6)	50.7 (9.3)	49.0 (9.8)
Age range, years	19.5 to 70.0	19.5 to 70.0	20.0 to 70.0	19.9 to 70.0	20.1 to 69.2	19.9 to 70.0
BMI, kg/m^2^, mean (SD)	25.8 (4.3)	26.6 (3.6)	25.4 (4.5)	26.0 (4.2)	26.6 (3.6)	25.7 (4.5)
Waist, cm, mean (SD)	84.6 (12.9)	94.6 (10.0)	79.8 (11.3)	86.0 (12.5)	94.0 (10.2)	80.8 (11.0)
Height, cm, mean (SD)	165.8 (9.1)	174.3 (7.3)	161.7 (6.7)	167.3 (9.6)	175.5 (7.3)	162.1 (6.9)
Postmenopausal status, %			49.5			41.8
Ever use of hormone for menopause, %			26.0			23.8
Smoking status, %						
Smoker	23.5	31.3	19.7	27.0	32.3	23.5
Former	27.4	36.2	23.1	27.8	37.3	21.6
Never	49.1	32.4	57.2	45.3	30.4	54.9
Physical activity, %						
Inactive	19.1	29.4	14.2	18.3	25.3	13.7
Moderately inactive	30.3	32.9	29.0	27.2	30.0	25.4
Moderately active	40.2	29.3	45.5	43.5	33.7	49.9
Active	10.3	8.5	11.2	11.0	11.0	11.0
Education, %						
None	5.9	4.9	6.3	3.1	2.4	3.6
Primary school completed	27.0	30.6	25.2	29.2	24.8	32.1
Technical school/professional school	22.4	22.7	22.3	31.1	29.8	31.9
Secondary school	16.2	11.9	18.3	13.4	11.4	14.7
University degree	24.6	27.4	23.3	23.0	31.5	17.5
Not specified	4.0	2.5	4.7	0.2	0.2	0.3
Dietary intake, g/day, median (IQR)						
Alcohol	6.6 (1.1 to 17.1)	14.4 (5.0 to 32.3)	4.1 (0.6 to 11.9)	7.8 (1.5 to 19.7)	16.4 (6.7 to 32.9)	4.2 (0.6 to 11.8)
Vegetables	196.9 (124.4 to 301.8)	179.7 (110.8 to 287.8)	204.9 (131.5 to 307.4)	130.0 (92.8 to 183.2)	124.0 (88.1 to 174.7)	133.9 (95.9 to 189.1)
Fruits	215.1 (117.1 to 340.8)	176.6 (88.5 to 313.3)	232.7 (132.4 to 351.8)	161.0 (94.1 to 264.7)	127.4 (73.9 to 225.3)	186.0 (105.6 to 290.3)
Dark bread	28.6 (0.0 to 91.8)	34.9 (0.0 to 112.5)	27.9 (0.0 to 87.8)	91.4 (15.0 to 150.2)	115.8 (49.3 to 179.6)	73.2 (8.0 to 128.0)
Dairy products	283.0 (159.5 to 447.5)	257.3 (130.0 to 434.3)	295.2 (173.1 to 451.7)	265.1 (150.0 to 434.7)	256.8 (136.7 to 447.0)	270.2 (158.8 to 428.7)
Red meat	38.2 (17.3 to 65.4)	49.7 (24.8 to 80.5)	33.8 (13.9 to 57.3)	44.8 (23.9 to 74.5)	61.9 (33.5 to 93.1)	36.9 (19.8 to 61.1)
Poultry	16.1 (5.8 to 30.9)	16.4 (7.3 to 34.3)	15.8 (4.8 to 29.3)	13.2 (6.5 to 24.3)	14.6 (7.3 to 25.5)	12.4 (5.9 to 23.4)
Processed meat	19.2 (6.4 to 37.9)	27.9 (10.8 to 51.9)	16.4 (5.4 to 31.8)	34.5 (17.9 to 59.6)	47.6 (27.4 to 76.7)	27.8 (14.6 to 48.7)
Fish	21.4 (9.3 to 37.0)	24.3 (12.6 to 41.2)	19.7 (7.7 to 34.9)	16.1 (6.0 to 30.3)	17.6 (6.3 to 32.2)	15.3 (5.9 to 27.9)
Sugar and confectionary	31.6 (16.3 to 55.2)	36.9 (19.2 to 65.0)	29.6 (15.2 to 51.0)	35.3 (18.7 to 60.0)	41.0 (21.7 to 70.3)	32.3 (17.1 to 54.1)
Soft drinks	9.5 (0.0 to 85.7)	16.4 (0.0 to 100.0)	6.6 (0.0 to 74.3)	8.7 (0.0 to 85.7)	19.3 (0.0 to 118.7)	3.8 (0.0 to 66.0)

*BMI* body mass index, *IQR* interquartile range, *SD* standard deviation

### Model development

**Fig. 2:**
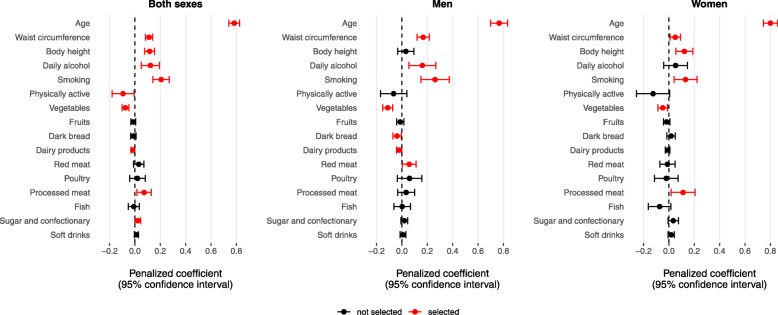
Average Cox regression coefficients with 95% confidence intervals after bootstrapped elastic net regularization. Bootstrapping was performed over 1000 repetitions. Selected variables with a confidence interval not including 0 are highlighted in red

Figure 2 illustrates the distribution of Cox regression coefficients of all predictor variables based on the bootstrapped elastic net regularization. Selected variables in the reduced model are highlighted based on the selection criterion of having a coefficient value of 0 not included in the 95% confidence interval. Table 2 shows derived colorectal cancer hazard ratios for all risk factors (full model) and risk factors that remained after elastic net selection (reduced model). The selected predictors of the overall colorectal cancer risk in men and women included age, waist circumference, height, daily alcohol consumption, smoking, physical activity, vegetables, dairy products, processed meat, and sugar and confectionary (Table 2). The models derived separately for men and women confirmed age, waist circumference, smoking and vegetable intake as consistent predictors across both genders. Additional predictors retained in the reduced model in men were daily alcohol consumption, dairy intake, dark bread and red meat, and in women, height and processed meat. The estimated 10-year mean absolute risk for colorectal cancer of the derivation cohort was 0.78% in both sexes, 1.07% in men and 0.64% in women (Table 2). Table 3 provides an overview of selected variables by anatomical subsite, colon and rectal cancer, overall and separately in men and women. An additional predictor that was retained in the model for rectal cancer was the intake of soft drinks. Notably, selected predictors in women were somewhat different for colon and rectal cancer. For colon cancer, the model included age, waist circumference, height, smoking and vegetable intake, whereas for rectal cancer it included age, processed meat and soft drinks (Table 3).

**Table 2:** Characteristics of colorectal cancer prediction models developed in the derivation cohort, overall and by sex

Predictor variables	Hazard ratios (95% confidence intervals), *p* value											
	Both sexes				Men				Women			
	Full model*		Reduced model† (LiFeCRC score)		Full model*		Reduced model†		Full model*		Reduced model†	
Age at recruitment, per 10 years	2.23 (2.13 to 2.33)	< .001	2.18 (2.09 to 2.29)	< .001	2.19 (2.04 to 2.35)	< .001	2.12 (1.98 to 2.27)	< .001	2.26 (2.13 to 2.40)	< .001	2.21 (2.08 to 2.34)	< .001
Waist circumference, per 10 cm	1.12 (1.09 to 1.15)	< .001	1.12 (1.09 to 1.16)	< .001	1.19 (1.13 to 1.25)	< .001	1.19 (1.13 to 1.25)	< .001	1.06 (1.01 to 1.10)	0.008	1.05 (1.01 to 1.09)	0.023
Height, per 10 cm	1.13 (1.08 to 1.17)	< .001	1.12 (1.08 to 1.17)	< .001	1.04 (0.96 to 1.11)	0.333			1.14 (1.06 to 1.22)	< .001	1.16 (1.08 to 1.24)	< .001
Daily alcohol consumption, high	1.14 (1.06 to 1.23)	< .001	1.14 (1.06 to 1.22)	< .001	1.18 (1.07 to 1.31)	0.002	1.18 (1.06 to 1.30)	0.002	1.06 (0.96 to 1.18)	0.245		
Ever smoker, yes	1.24 (1.16 to 1.33)	< .001	1.24 (1.15 to 1.32)	< .001	1.31 (1.17 to 1.47)	< .001	1.31 (1.17 to 1.46)	< .001	1.15 (1.05 to 1.26)	0.003	1.16 (1.06 to 1.27)	0.001
Physically active, yes	0.89 (0.82 to 0.97)	0.010	0.91 (0.83 to 0.99)	0.026	0.93 (0.83 to 1.04)	0.185			0.87 (0.76 to 0.99)	0.029		
Vegetables, per 100 g/day	0.92 (0.90 to 0.95)	< .001	0.93 (0.90 to 0.95)	< .001	0.89 (0.85 to 0.93)	< .001	0.89 (0.85 to 0.92)	< .001	0.95 (0.92 to 0.99)	0.009	0.93 (0.90 to 0.97)	< .001
Fruits, per 100 g/day	0.99 (0.97 to 1.01)	0.180			0.99 (0.96 to 1.02)	0.424			0.98 (0.96 to 1.01)	0.196		
Dark bread, per 50 g/day	0.98 (0.96 to 1.01)	0.147			0.96 (0.93 to 0.99)	0.015	0.97 (0.94 to 1.00)	0.070	1.02 (0.98 to 1.06)	0.329		
Dairy products, per 100 g/day	0.98 (0.97 to 0.99)	0.006	0.98 (0.97 to 1.00)	0.017	0.98 (0.96 to 1.00)	0.016	0.98 (0.96 to 1.00)	0.048	0.99 (0.97 to 1.01)	0.198		
Red meat, per 50 g/day	1.03 (0.99 to 1.08)	0.158			1.06 (1.00 to 1.13)	0.046	1.08 (1.02 to 1.14)	0.010	0.98 (0.91 to 1.06)	0.577		
Poultry, per 50 g/day	1.03 (0.95 to 1.12)	0.461			1.07 (0.96 to 1.20)	0.210			0.99 (0.87 to 1.12)	0.845		
Processed meat, per 50 g/day	1.08 (1.02 to 1.14)	0.006	1.08 (1.03 to 1.14)	0.004	1.04 (0.97 to 1.11)	0.296			1.13 (1.03 to 1.24)	0.010	1.12 (1.02 to 1.23)	0.020
Fish, per 50 g/day	0.99 (0.93 to 1.05)	0.665			1.00 (0.93 to 1.09)	0.914			0.92 (0.83 to 1.02)	0.109		
Sugar and confectionary, per 50 g/day	1.03 (1.00 to 1.05)	0.028	1.03 (1.00 to 1.05)	0.022	1.02 (0.99 to 1.05)	0.118			1.04 (0.99 to 1.09)	0.088		
Soft drinks, per 100 g/day	1.02 (1.00 to 1.03)	0.097			1.01 (0.99 to 1.04)	0.392			1.02 (1.00 to 1.05)	0.095		
Survival function_*m*_ (10 years)‡	0.9944		0.9943		0.9920		0.9919		0.9953		0.9952	
Risk Score_*m*_§	6.8953		6.8089		6.2600		5.5356		6.4758		6.7039	
Absolute Risk_*m*_ (10 years)#	0.78%		0.78%		1.07%		1.07%		0.64%		0.64%	

*Full models were derived by using all available predictor variables

†Reduced models were derived by using bootstrapped elastic net variable selection with all predictor variables of the full model

‡Survival function_*m*_ (10 years): 10-year survival function estimate of average predictor values of the derivation cohort. Estimates for timespans between 0 and 20 years are shown in Supplementary Fig. 2, Additional File 2

§Risk Score_*m*_: Mean risk score calculated based on the sum of beta coefficient products of average predictor values of the derivation cohort

#Absolute Risk_*m*_: 10-year mean absolute risk of the derivation cohort

**Table 3:** Characteristics of colon and rectal cancer prediction models developed in the derivation cohort, overall and by sex

Predictor variables	Hazard ratios (95% confidence intervals), p-value											
	Colon cancer						Rectal cancer					
	Both sexes		Men		Women		Both sexes		Men		Women	
Age at recruitment, per 10 years	2.30 (2.17 to 2.44)	< .001	2.11 (1.93 to 2.30)	< .001	2.31 (2.15 to 2.49)	< .001	2.02 (1.87 to 2.18)	< .001	2.10 (1.88 to 2.35)	< .001	1.86 (1.68 to 2.04)	< .001
Waist circumference, per 10 cm	1.14 (1.10 to 1.18)	< .001	1.27 (1.19 to 1.35)	< .001	1.07 (1.02 to 1.12)	0.006	1.10 (1.05 to 1.15)	< .001				
Height, per 10 cm	1.14 (1.08 to 1.19)	< .001			1.17 (1.08 to 1.28)	< .001	1.15 (1.08 to 1.23)	< .001				
Daily alcohol consumption, high	1.12 (1.02 to 1.22)	0.017					1.21 (1.07 to 1.37)	0.002	1.27 (1.08 to 1.50)	0.004		
Ever smoker, yes	1.18 (1.08 to 1.28)	< .001	1.18 (1.02 to 1.35)	0.024	1.14 (1.02 to 1.28)	0.018	1.32 (1.17 to 1.49)	< .001	1.44 (1.19 to 1.74)	< .001		
Physically active, yes	0.88 (0.80 to 0.98)	0.025			0.84 (0.72 to 0.99)	0.035						
Vegetables, per 100 g/day	0.91 (0.89 to 0.94)	< .001	0.90 (0.85 to 0.94)	< .001	0.93 (0.89 to 0.97)	0.001	0.92 (0.88 to 0.96)	< .001	0.88 (0.82 to 0.93)	< .001		
Fruits, per 100 g/day												
Dark bread, per 50 g/day	0.96 (0.93 to 0.99)	0.017	0.94 (0.90 to 0.98)	0.008								
Dairy products, per 100 g/day	0.98 (0.96 to 0.99)	0.007	0.96 (0.93 to 0.98)	0.001								
Red meat, per 50 g/day												
Poultry per, 50 g/day												
Processed meat, per 50 g/day							1.20 (1.10 to 1.31)	< .001			1.27 (1.09 to 1.47)	0.002
Fish, per 50 g/day												
Sugar and confectionary, per 50 g/day												
Soft drinks, per 100 g/day							1.03 (1.00 to 1.06)	0.025			1.06 (1.02 to 1.10)	0.006
Survival function_*m*_ (10 years)*	0.9966		0.9953		0.9970		0.9979		0.9968		0.9982	
Risk Score_*m*_†	7.156		5.802		7.137		6.8764		3.9117		3.2985	
Absolute Risk_*m*_ (10 years)‡	0.48%		0.62%		0.41%		0.28%		0.42%		0.21%	

*Survival function_*m*_ (10 years): 10-year survival function of average predictor values of the derivation cohort

†Risk Score_*m*_: Mean risk score calculated based on the sum of beta coefficient products of average predictor values of the derivation cohort

‡Absolute Risk_*m*_: 10-year mean absolute risk of the derivation cohort

### Model performance: discrimination and calibration

**Fig. 3:**
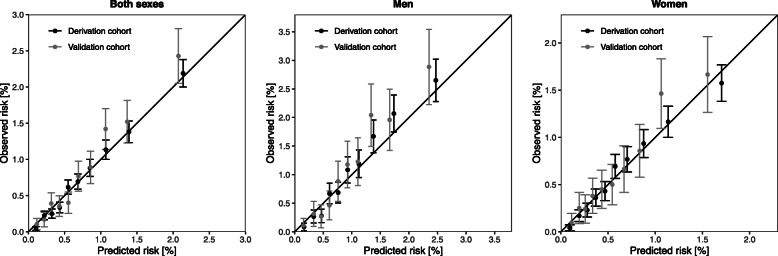
Calibration plots of 10-year colorectal cancer risk. Predicted risk is compared against observed risk in the derivation and validation cohorts, overall and by sex. Observed risk is based on the complement of the Kaplan-Meier survival curve

Overall model discrimination was good with Harrell’s C-index of 0.709 for the derived colorectal cancer risk model. Optimism-adjusted Harrell's C index  ranged from 0.667 for the model for rectal cancer in women to 0.716 for the model for colon cancer in both sexes (Table 4). Reduced models showed similar predictive performance as the “full models” suggesting that obtaining data on selected predictors would yield sufficient information and additional factors are not adding predictive value to the model. The performance in the validation cohort was similar for all models, suggesting a high level of stability and a lack of overfitting. Calibration plots of derived colorectal cancer risk models in the derivation and validation sample overall and by sex are presented in Fig. 3. An overall good calibration was observed based on the comparable intercepts for models across derivation and validation samples.

**Table 4:** Model selection and discrimination in the derivation and validation cohorts

	Colorectal cancer			Colon cancer			Rectal cancer		
Selected predictors	Both sexes	Men	Women	Both sexes	Men	Women	Both sexes	Men	Women
Age at recruitment, per 10 years	●	●	●	●	●	●	●	●	●
Waist circumference, per 10 cm	●	●	●	●	●	●	●		
Height, per 10 cm	●		●	●		●	●		
Daily alcohol consumption, high	●	●		●			●	●	
Ever smoker, yes	●	●	●	●	●	●	●	●	
Physically active, yes	●			●		●			
Vegetables, per 100 g/day	●	●	●	●	●	●	●	●	
Fruits, per 100 g/day									
Dark bread, per 50 g/day		●		●	●				
Dairy products, per 100 g/day	●	●		●	●				
Red meat, per 50 g/day		●							
Poultry, per 50 g/day									
Processed meat, per 50 g/day	●		●				●		●
Fish, per 50 g/day									
Sugar and confectionary, per 50 g/day	●								
Soft drinks, per 100 g/day							●		●
**Harrell’s C-index**									
Full model									
Derivation cohort	0.710	0.700	0.702	0.718	0.708	0.718	0.705	0.705	0.677
Optimism corrected *	0.708	0.697	0.700	0.716	0.707	0.715	0.704	0.703	0.668
Validation cohort	0.715	0.707	0.700	0.708	0.727	0.700	0.730	0.689	0.693
Reduced model									
Derivation cohort	0.710	0.699	0.700	0.717	0.705	0.717	0.703	0.700	0.668
Optimism corrected*	0.709	0.698	0.699	0.716	0.704	0.715	0.701	0.698	0.667
Validation cohort	0.714	0.708	0.699	0.708	0.727	0.698	0.728	0.687	0.696

*Harrell's C-index for the derivation cohort corrected for optimism by bootstrapping with 1000 replications. For each bootstrap sample a new model is fitted and the C-index calculated for the bootstrap sample and the original derivation cohort. The difference between these two C-indices is then averaged over all bootstrap replications and then subtracted from the original C-index

### Model communication

#### Absolute risk formula

To provide assessment of the absolute 10-year risk  of colorectal cancer for individuals with various combinations of risk factors, we prepared a formula with the following selected predictors:

$$ \mathrm{Absolute}\ \mathrm{risk}\kern0.28em \left(\begin{array}{l}\mathrm{Colorectal}\kern0.34em \mathrm{cancer}\\ {}\mathrm{within}\kern0.28em 10\kern0.28em \mathrm{years}\end{array}\right)=1-{S}_m{\left(10\kern0.28em \mathrm{years}\right)}^{\exp \left(\mathrm{Risk}\kern0.28em {\mathrm{Score}}_i-\mathrm{Risk}\kern0.28em {\mathrm{Score}}_m\right)}=1-{0.9943}^{\exp \left(\mathrm{Risk}\kern0.28em {\mathrm{Score}}_i-6.8089\right)} $$

$$ {\mathrm{Risk}\ \mathrm{Score}}_i=0.0781\times {\mathrm{Age}}_i\kern0.20em \left(\mathrm{years}\right)+0.0117\times {\mathrm{Waist}\ \mathrm{circumference}}_i\kern0.20em \left(\mathrm{cm}\right)+0.0115\times {\mathrm{Body}\ \mathrm{height}}_i\kern0.20em \left(\mathrm{cm}\right)+0.1292\times {\mathrm{Daily}\ \mathrm{alcohol}}_i\kern0.20em \left(\mathrm{yes}=1,\mathrm{no}=0\right)+0.2125\times {\mathrm{Smoking}}_i\kern0.20em \left(\mathrm{yes}=1,\mathrm{no}=0\right)-0.0964\times {\mathrm{Physically}\ \mathrm{active}}_i\kern0.20em \left(\mathrm{yes}=1,\mathrm{no}=0\right)-0.0773\times {\mathrm{Vegetable}\ \mathrm{intake}}_i\kern0.20em \left(\mathrm{per}\kern0.20em 100\mathrm{g}/\mathrm{day}\right)-0.0166\times \mathrm{Dairy}\ {\mathrm{products}\ \mathrm{intake}}_i\kern0.20em \left(\mathrm{per}\kern0.20em 100\mathrm{g}/\mathrm{day}\right)+0.0808\times \mathrm{Processed}\ {\mathrm{meat}\ \mathrm{intake}}_i\kern0.20em \left(\mathrm{per}\kern0.20em 50\mathrm{g}/\mathrm{day}\right)+0.0268\times \mathrm{Sugar}\ {\mathrm{and}\ \mathrm{confectionary}}_i\kern0.20em \left(\mathrm{per}\kern0.20em 50\mathrm{g}/\mathrm{day}\right) $$

Values for *S*_*m*_ (10 years) and Risk Score_*m*_ are given in Table 2. Absolute risk for different timespans can be calculated by replacing *S*_*m*_ in the formula accordingly. The survival function estimates for timespans between 0 and 20 years are shown in Supplementary Fig. 2, Additional File 2. Incidence rates and model selection characteristics across predefined risk categories (low, intermediate and high risk) with cut points at 0.62% and 1.60% 10-year absolute risk are presented in Supplementary Table 4, Additional File 1, for both the derivation and validation sample.

#### Nomogram

**Fig. 4:**
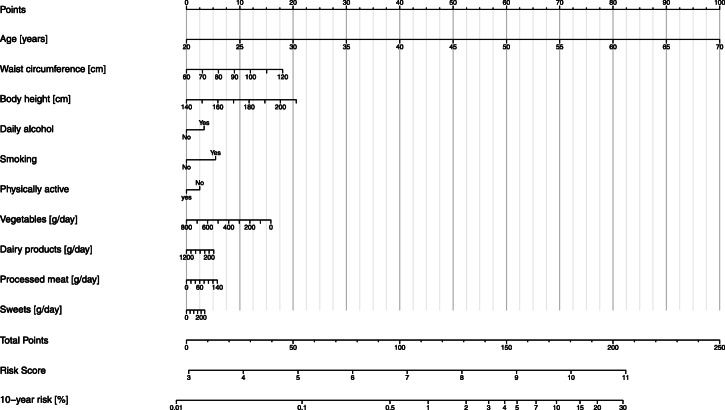
Nomogram of colorectal cancer absolute risk prediction over 10 years

Figure 4 shows a nomogram of the weights and points of the colorectal cancer risk prediction score allowing estimation of an individual’s probability to develop colorectal cancer over a 10-year period. The nomogram is characterized by a scale corresponding to each variable, a point scale, a total point scale and a probability scale*.* The use of the nomogram is simple and involves 3 steps. First, on the scale for each variable, the value corresponding to a specific individual is read and the point scale is used to calculate the points for all variable values. Second, the total number of points is calculated by adding up all the points obtained in the previous step, and its value is identified on the total point scale. Finally, the probability of an event corresponding to the total points of the individual is represented on the risk scale. As a practical example, we estimated the 10-year risk of colorectal cancer, for individuals with two different combinations of ages and lifestyle factors, representing low-risk and high-risk extremes: individual 1 was 45 years old (50 points) with a body height of 166 cm (7.5 points), a waist circumference of 70 cm (3 points) and healthy lifestyle behaviour (low daily alcohol consumption (0 points), non-smoker (0 points), physically active (0 points), 430 g daily vegetable intake (7 points), 630 g daily dairy products intake (2.5 points), 0 g daily processed meat intake (0 points), and 5 g daily sugar and confectionary intake (0 points)), and individual 2 was 65 years old (90 points) with a body height of 166 cm (7.5 points), a waist circumference of 100 cm (12 points) and rather unhealthy lifestyle behaviour (high daily alcohol consumption (3 points), smoker (5 points), physically inactive (2.5 points), 80 g daily vegetable intake (14.5 points), 70 g daily dairy products intake (5 points), 60 g daily processed meat intake (2.5 points), and 90 g daily sugar and confectionary intake (1.5 points)). The total number of points of the various prediction indicators was ~ 70 and ~ 143.5 and the corresponding absolute predicted 10-year risk of colorectal cancer was ~ 0.2% (risk score of ~ 5.7) and ~ 3–3.5% (risk score of ~ 8.6) for individual 1 and individual 2, respectively.

#### Web-based calculator

**Fig. 5:**
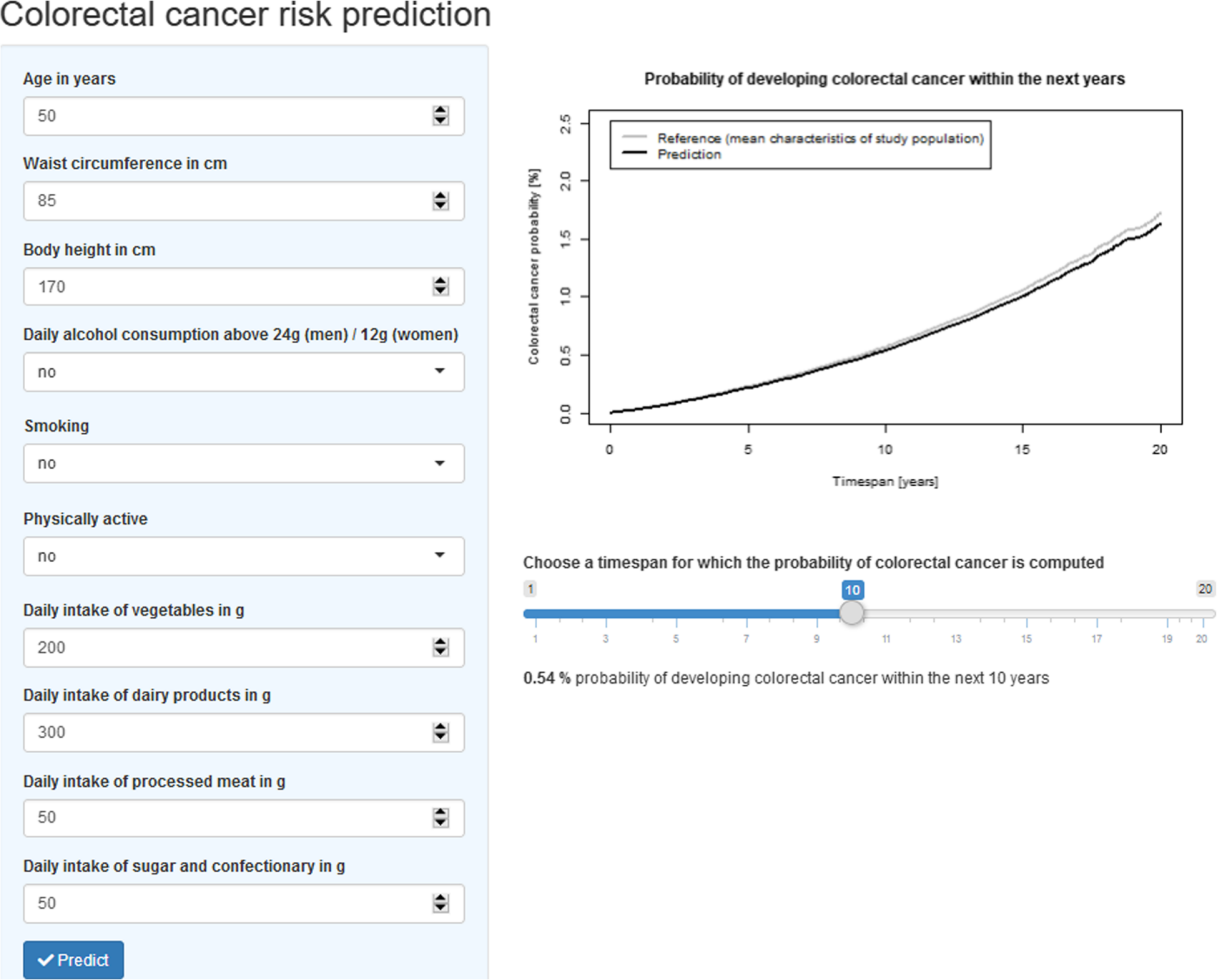
Application for the colorectal cancer risk model. Example for a hypothetical individual data entry and risk calculation

As an alternative approach to model communication, we developed a web-based calculator for the estimation of a personalized colorectal cancer risk based on the validated LiFeCRC score. A graphical illustration of the application layout with predicted and absolute risk values for a modifiable time span is presented in Fig. 5. Of note, the results produced by the web-based calculator should be interpreted considering that competing risk of mortality was not included in the absolute risk calculation.

### Random survival forest

Results of random survival forest-based relative variable importance for colorectal cancer risk prediction are presented in Supplementary Fig. 3, Additional File 2. The main selected predictors remained similar as in the Cox regression model, confirming model robustness. The highest relative importance was observed for age, followed by waist circumference, red and processed meat intake, height and vegetable consumption. The model for women showed, in addition, height, dark bread and dairy products intake as additional important predictors, whereas the model for men showed smoking and sweets and confectionary consumption as additional important predictors. Overall, the discrimination (Supplementary Fig. 3, Additional File 2) and calibration (Supplementary Fig. 4, Additional File 2) of the random survival forest based colorectal cancer risk prediction model was comparable to the Cox regression model.

### Sensitivity analysis

In a sensitivity analysis, we evaluated to what extent lifestyle data added predictive value to the colorectal cancer risk model based on age only. The addition of the lifestyle variables resulted in a statistically significantly increased goodness of fit (likelihood ratio test *p* < 0.001). The estimated NRI^> 0^ was 0.307 (95% confidence interval 0.264 to 0.352) indicating an improvement in model performance. Supplementary Fig. 5, Additional File 2 displays the model calibration and net benefit curves for an aged-based model and the LiFeCRC model that additionally included lifestyle factors for overall colorectal cancer. An improved calibration and higher net benefit were observed for colorectal cancer risk thresholds between 0.7 and 2.5% for the LiFeCRC model compared to the age-based model. In analyses stratified according to age groups, model performance was higher in individuals < 45 years and adding lifestyle data contributed to improved reclassification statistics, i.e. higher NRI^> 0^, suggesting relative importance of lifestyle data assessment for risk prediction at younger ages (< 45 years), i.e. NRI^> 0^ = 0.364 (95% confidence interval 0.084 to 0.575) (Supplementary Table 5, Additional File 1). We further estimated the predicted 10-year absolute risk of colorectal cancer for an arbitrary predefined “healthy” and “unhealthy” lifestyle, across different age groups and a constant body height (Supplementary Fig. 6, Additional File 2). For example, an individual aged 45 years with a body height of 166 cm adopting a predefined “unhealthy lifestyle” (waist circumference of 100 cm, high daily alcohol consumption, smoker, physically inactive, 80 g daily vegetable intake, 70 g daily dairy products intake, 60 g daily processed meat intake and 90 g daily sugar and confectionary intake) has a 3.6 times higher absolute risk of colorectal cancer within the next 10 years compared to a person of the same age and body height, adopting a predefined “healthy lifestyle” (waist circumference of 70 cm, low daily alcohol consumption, non-smoker, physically active, 430 g daily vegetable intake, 630 g daily dairy products intake, 0 g daily processed meat intake and 5 g daily sugar and confectionary intake). In a subsample with available information, addition of information on NSAID use or family history of colorectal cancer to the list of predictors did not further improve model performance beyond main lifestyle variables (Supplementary Fig. 7, Additional File 2). The results did not reveal marked differences in model discrimination among subgroups by waist circumference, education, smoking status and levels of alcohol consumption (Supplementary Table 6, Additional File 1). Furthermore, no substantial differences could be seen between the Kaplan-Meier survival function and the cumulative incidence function taking competing risk into account (data not shown). Also, no differences in the discrimination ability of the Fine-Gray model taking competing risk of death into account could be observed (C-index = 0.710).

## Discussion

In this large European prospective cohort  study, we developed and validated the LiFeCRC score, as a lifestyle-based prediction model for the prevention of colorectal cancer in asymptomatic populations across Europe. Beyond age, the variables retained in the model were waist circumference, height, daily alcohol consumption, smoking status, physical activity and dietary intakes of vegetables, dairy products, processed meat and sugar and confectionary. Separate models were also developed for men and women and for colon and rectal cancer subtypes. The model showed good calibration and discrimination properties to identify individuals at all levels of colorectal cancer risk. Modifiable lifestyle factors contributed to model performance and accuracy beyond age alone and could improve reclassification statistics especially in younger age groups (< 45 years). A user-friendly colorectal cancer risk nomogram and a web calculator were developed to facilitate model communication.

Currently, the target population for colorectal cancer screening is mainly selected based on age alone (i.e. 50 years or above). Although age is undoubtedly an important predictor of colorectal cancer as shown in our data, information on modifiable lifestyle factors allows provision of preventive health recommendations for individuals at risk [40]. Lifestyle-based models have been suggested in medical practice as important tools that could be used to identify those most likely to benefit from lifestyle interventions and to contribute to behaviour change interventions [41]. A number of intervention studies focusing on changing lifestyle for colorectal cancer prevention reported significant effects on the target behaviours [42–46]. In those studies, tailored approaches that enable personalized feedback regarding individual lifestyle patterns were suggested as more successful compared to generic approaches [42–47]. Despite lifestyle interventions representing a powerful cost-effective strategy for colorectal cancer prevention, there has been little incentive on the side of health professionals to advocate lifestyle-based recommendations [48]. Risk assessment tools such as the LifeCRC score could facilitate improved advocacy on the side of health professionals and motivate or empower individuals to implement behaviour changes [47, 49]. Targeting lifestyle factors in those at highest risk may be particularly relevant for younger age groups that may profit most from early preventive interventions aimed at encouraging behavioural changes [47].

A number of previous models incorporated lifestyle data with common covariates including self-reported BMI (body mass index), alcohol consumption and smoking [18–21]. Recently, a model based on BMI, smoking, alcohol, red and processed meat, fruits, vegetables and physical activity demonstrated C-statistics of 0.66 and 0.68 in men and women, respectively [41]. Compared with this and other published models that also include family history and more complex variables [18, 19, 50, 51], the EPIC lifestyle-based model showed a comparable and even improved performance based on Harrell’s C-index of 0.710 in both derivation and validation cohort. As previously reported, the highest C-statistic for colorectal cancer risk prediction model ranged from 0.67 in UK Biobank to 0.69 EPIC validation samples [20]. Compared to our model, that model included 13 variables: age, ethnicity, education, BMI, family history, diabetes, oestrogen exposure, non-steroidal anti-inflammatory use, physical activity, smoking, alcohol, red meat intake and multivitamin use. Having the strong discrimination statistics for models based on age alone, additional predictors were shown to add little improvement to model C-statistics in previous studies as well as in our data [18, 20, 51]. To address the question whether lifestyle information is important for absolute risk assessment beyond age, we evaluated the model performance across different age groups. These results showed that the model performance was highest in the group of participants < 45 years old and suggested this age period as a relevant time window for early cancer prevention. We further calculated the 10-year absolute risk of colorectal cancer across different ages comparing predefined “healthy” versus “unhealthy” lifestyle pattern based on selected model predictors. These analyses suggested that at a given age and height, i.e. for an individual aged 45 years with a body height of 166 cm, following the unhealthy lifestyle pattern would lead to 3.6 times higher absolute risk of colorectal cancer within the next 10 years compared to a person of the same age and body height, adopting a healthy lifestyle. These results highlight the importance of adherence to healthy lifestyle for the long-term reduction of colorectal cancer risk. In support of these data, recent analysis based on a large German population sample showed that healthy lifestyle could improve prospects for avoiding colorectal cancer in the long term even beyond individual genetic risk [52].

The elaborated phenotyping and detailed assessment of nutritional data in the EPIC cohort allowed selection of several factors not commonly depicted in previous colorectal cancer risk prediction models. Compared to previous models that used data on self-reported BMI, in the EPIC cohort data was available on waist circumference measurements and these were among the main predictors [53, 54]. Unlike BMI which does not take body fat distribution into account, waist circumference provides a proxy for the centrally located visceral fat shown especially relevant for colorectal cancer development [53, 55]. Only a few previous models included data on height which was selected as another important predictor by our model [56, 57]. Greater height could provide reflection of an increased standard of living characterized by greater availability of energy and protein-rich foods, lower physical activity and a reduced incidence of childhood infections that follow different patterns across Europe [58]. Physical activity was also selected as a predictor of colorectal cancer risk, particularly in the model for women. These data support recent findings from the Women’s Health Initiative [59] and the overall notion of the importance of physical activity for the prevention of colorectal cancer [60]. Beyond red meat [56, 57, 61] and vegetable intake [56, 62–64], additional dietary predictors selected by our model included low dairy intake and high intakes of sugary products, including soft drinks. Guiding individuals towards healthy dietary and lifestyle choices could complement colorectal cancer screening as means for colorectal cancer prevention.

The selected model performed similarly well as the model with the full list of predictors, suggesting that it can be used as a simpler approach for determining high-risk individuals. Thus, individuals and health professionals would need to inquire about fewer lifestyle factors, avoiding the use of long questionnaires and minimizing the burden of data collection on both the patient and clinician side. However, for a comprehensive lifestyle recommendation, all healthy behaviours could be considered in additional counselling. The model performance among women was modest, and better in men, likely because some risk factors were more strongly associated with risk among men. The general distribution and influence of risk factors may differ geographically across populations and additional model elaboration and adaptation of country-specific risk models should be further considered. Ultimately, research is needed to assess the feasibility and effectiveness of the current lifestyle-based risk assessment tool on health behaviour modification, colorectal cancer risk factor improvement, and overall potential for colorectal cancer prevention when incorporated into the primary care setting, particularly as a pre-screening instrument of high-risk patients. More work is also warranted for the refinement of the risk communication tool before its general integration into practice. Finally, in future research, additional predictors, including relevant biomarker and genetic variables, should be further explored on the way towards improved precision prevention of colorectal cancer. For example, in a systematic review of 29 studies, addition of common single nucleotide polymorphisms (SNPs) to other risk factors in models developed in asymptomatic individuals in the general population increased model discrimination by 0.01 to 0.06 [19]. Overall, the reported C-statistic ranged from 0.56 to 0.63 for SNPs alone and in combination with other risk factors, respectively [19]. Further studies are warranted to evaluate whether employing genetic risk profiling beyond established risk factors can be useful to identify individuals at high colorectal cancer risk.

Our work has several strengths. The EPIC study provided an ideal setting to develop a lifestyle-based colorectal cancer risk prediction model, given its large sample size, various population backgrounds and a long follow-up time of over 20 years. Furthermore, the study provided a variety of objectively measured anthropometric data along with dietary and lifestyle information. Therefore, the current model is the first developed on a European-wide study population sample, allowing assessment of risk across a broad range of diet and lifestyle behaviours. Given the large sample size, we were also able to validate the risk scores in an independent subset of the EPIC populations. Additionally, we derived the colorectal cancer risk estimates empirically following state-of-the-art and novel machine learning approaches, i.e. random survival forest, considering various predictors simultaneously and the gradient in risk across the full distribution of risk levels. Finally, we considered model application and suggested a nomogram and a web-tool to enable risk communication. Several potential limitations of our study warrant discussion. First, we derived the risk equations based on a study population comprising of volunteers. Volunteer-based studies are prone to include individuals who are often more likely to have favourable exposure and health profiles compared to those who do not. Thus, higher prevalence of healthy behaviours in our sample as compared to the general population could have resulted in overestimated absolute risk estimates. Second, with the exception of age and the anthropometric measures, we relied on data of self-reported predictors and routinely collected cancer outcomes. Though any risk prediction tool made publicly available online would also rely on self-reported data, more accurate risk factor ascertainment would possibly improve overall model discrimination and calibration. Nevertheless, our model has shown a good discrimination and excellent calibration. Third, dietary data was collected using food frequency questionnaires as a commonly applied dietary assessment method in epidemiology, however future model application should consider further adaptation and feasibility assessment to facilitate model communication in practice. Fourth, we based analyses on lifestyle information collected at study baseline and, therefore, could not account for potential behavioural changes during study follow-up. Finally, the model was developed based on data available in the EPIC cohort and did not include some potentially important predictors, such as NSAID use or family history of colorectal cancer. However, we have conducted a sensitivity analysis using data from study centres that collected these data and the model performance was not altered.

## Conclusions

Despite being one of the leading causes of cancer morbidity and mortality, colorectal cancer is largely preventable. LiFeCRC score based on age and lifestyle data accurately identifies individuals at risk for incident colorectal cancer in European populations and could contribute to improved prevention through motivating lifestyle change at the individual level.

## Supplementary information

**Additional file 1:** **Supplementary Table 1.** Baseline characteristics of participants with available information on NSAID use and colorectal cancer family history. **Supplementary Table 2.** TRIPOD Checklist - Prediction Model Development and Validation. **Supplementary Table 3.** Factors considered for inclusion in the LiFeCRC score. **Supplementary Table 4.** LifeCRC model selection characteristics across pre-defined risk categories in the derivation and validation samples. **Supplementary Table 5.** Added predictive performance for age and lifestyle-based (LiFeCRC) colorectal cancer risk prediction models. Results are stratified by age groups. **Supplementary Table 6.** Sensitivity analysis by subgroups.

**Additional file 2: Supplementary Figure 1.** Workflow of risk model development and validation. **Supplementary Figure 2.** Survival function of average predictor values of the derivation cohort. **Supplementary Figure 3.** Discrimination and relative variable importance based on Random Survival Forest models for colorectal cancer prediction. **Supplementary Figure 4.** Random Survival Forest colorectal cancer full model calibration. **Supplementary Figure 5.** Model performance comparison the LiFeCRC score and a colorectal cancer risk model including only age. (a) Calibration plot of predicted 10-year colorectal cancer risk for a model that included only age and the LiFeCRC score model with additional lifestyle predictors (waist circumference, body height, daily alcohol consumption, smoking, physical activity, and daily intake of vegetables, dairy products and red meat). (b) Decision curves illustrating net benefit of prediction models for a range of colorectal cancer risk thresholds, used to decide about further treatment or intervention. Decisions curves are shown for different models: none treatment, all treatment, treatment based on the age-model, treatment based on the LiFeCRC model. **Supplementary Figure 6.** Predicted 10-year absolute risk of colorectal cancer for a healthy and unhealthy lifestyle. Risk across different age-groups and a constant body height of 166 cm. *Unhealthy lifestyle:* waist circumference of 100 cm, high daily alcohol consumption, smoker, physically inactive, 80 g daily vegetable intake, 70 g daily dairy products intake, 60 g daily processed meat intake, and 90 g daily sugar and confectionary intake. *Healthy lifestyle:* waist circumference of 70 cm, low daily alcohol consumption, non-smoker, physically active, 430 g daily vegetable intake, 630 g daily dairy products intake, 0 g daily processed meat intake, and 5 g daily sugar and confectionary intake. **Supplementary Figure 7.** Full model performance including NSAID use and family history of colorectal cancer.

## Data Availability

EPIC data are available for investigators who seek to answer important questions on health and disease in the context of research projects that are consistent with the legal and ethical standard practices of IARC/WHO (World Health Organization) and the EPIC centres. The primary responsibility for accessing the data belongs to the EPIC centres that provided them. For information on how to submit an application for gaining access to EPIC data and/or biospecimens, please follow the instructions at http://epic.iarc.fr/access/index.php.

## Abbreviations

AICR: American Institute for Cancer Research
BMI: Body mass index
C-index: Harrell’s concordance index
CRC: Colorectal cancer
EPIC: European Prospective Investigation into Cancer and Nutrition
ICD: International Statistical Classification of Diseases
IQR: Interquartile range
LASSO: Least absolute shrinkage and selection operator
LiFeCRC score: Lifestyle-based prediction model for colorectal cancer risk
NRI^> 0^: Continuous net reclassification improvement
NSAID: Non-steroidal anti-inflammatory drug
RSF: Random survival forest
SD: Standard deviation
SNPs: Single nucleotide polymorphisms
TRIPOD: Transparent Reporting of a multivariable Prediction model for Individual Prognosis or Diagnosis
UK: United Kingdom
WCRF: World Cancer Research Fund
