# Supplementary material for: Development and validation of a lifestyle-based model for colorectal cancer risk prediction: the LiFeCRC score
Source: BMC Med. 2021 Jan 4;19:1. doi: 10.1186/s12916-020-01826-0 (PMC7780676; doi:10.1186/s12916-020-01826-0)
Supplement: Supplementary file 1 — Additional file 1: Supplementary Table 1. Baseline characteristics of participants with available information on NSAID use and colorectal cancer family history. Supplementary Table 2. TRIPOD Checklist - Prediction Model Development and Validation. Supplementary Table 3. Factors considered for inclusion in the LiFeCRC score. Supplementary Table 4. LifeCRC model selection characteristics across pre-defined risk categories in the derivation and validation samples. Supplementary Table 5. Added predictive performance for age and lifestyle-based (LiFeCRC) colorectal cancer risk prediction models. Results are stratified by age groups. Supplementary Table 6. Sensitivity analysis by subgroups. [file 12916_2020_1826_MOESM1_ESM.docx]

**Supplementary Table 1.** Baseline characteristics of participants with available information on NSAID use and colorectal cancer family history.

| **Characteristics** | **Sample with available information on NSAID use** |  | **Sample with available information on  Family history** |
| --- | --- | --- | --- |
| *N* | 14,541 |  | 60,296 |
| Population origin | Cambridge (United Kingdom) |  | France, Spain, United Kingdom |
| Age at recruitment, years, mean (SD) | 56.1 (7.6) |  | 51.9 (8.1 |
| Sex, % women | 54.8 |  | 69.4 |
| NSAID use, % |  |  |  |
| yes | 7.1 |  |  |
| no | 92.9 |  |  |
| Family history of colorectal cancer, % |  |  |  |
| yes |  |  | 7.4 |
| no |  |  | 92.6 |

**Supplementary Table 2.** TRIPOD Checklist - Prediction Model Development and Validation.

| **Section/Topic** | **Item** |  | **Checklist Item** | **Page** |
| --- | --- | --- | --- | --- |
| **Title and abstract** |  |  |  |  |
| Title | 1 | D;V | Identify the study as developing and/or validating a multivariable prediction model, the target population, and the outcome to be predicted. | 1 |
| Abstract | 2 | D;V | Provide a summary of objectives, study design, setting, participants, sample size, predictors, outcome, statistical analysis, results, and conclusions. | 5-6 |
| **Introduction** |  |  |  |  |
| Background and objectives | 3a | D;V | Explain the medical context (including whether diagnostic or prognostic) and rationale for developing or validating the multivariable prediction model, including references to existing models. | 7-8 |
|  | 3b | D;V | Specify the objectives, including whether the study describes the development or validation of the model or both. | 8 |
| **Methods** |  |  |  |  |
| Source of data | 4a | D;V | Describe the study design or source of data (e.g., randomized trial, cohort, or registry data), separately for the development and validation data sets, if applicable. | 8-10 |
|  | 4b | D;V | Specify the key study dates, including start of accrual; end of accrual; and, if applicable, end of follow-up. | 8-9 |
| Participants | 5a | D;V | Specify key elements of the study setting (e.g., primary care, secondary care, general population) including number and location of centres. | 8-10 |
|  | 5b | D;V | Describe eligibility criteria for participants. | 8-10 |
|  | 5c | D;V | Give details of treatments received, if relevant. | - |
| Outcome | 6a | D;V | Clearly define the outcome that is predicted by the prediction model, including how and when assessed. | 9 |
|  | 6b | D;V | Report any actions to blind assessment of the outcome to be predicted. | - |
| Predictors | 7a | D;V | Clearly define all predictors used in developing or validating the multivariable prediction model, including how and when they were measured. | 10-11 |
|  | 7b | D;V | Report any actions to blind assessment of predictors for the outcome and other predictors. | - |
| Sample size | 8 | D;V | Explain how the study size was arrived at. | 9-10 |
| Missing data | 9 | D;V | Describe how missing data were handled (e.g., complete-case analysis, single imputation, multiple imputation) with details of any imputation method. | 9-10 |
| Statistical analysis methods | 10a | D | Describe how predictors were handled in the analyses. | 11 |
|  | 10b | D | Specify type of model, all model-building procedures (including any predictor selection), and method for internal validation. | 11-13 |
|  | 10c | V | For validation, describe how the predictions were calculated. | 11-13 |
|  | 10d | D;V | Specify all measures used to assess model performance and, if relevant, to compare multiple models. | 11-14 |
|  | 10e | V | Describe any model updating (e.g., recalibration) arising from the validation, if done. | - |
| Risk groups | 11 | D;V | Provide details on how risk groups were created, if done. | 12-13 |
| Development vs. validation | 12 | V | For validation, identify any differences from the development data in setting, eligibility criteria, outcome, and predictors. | 10-11 |
| **Results** |  |  |  |  |
| Participants | 13a | D;V | Describe the flow of participants through the study, including the number of participants with and without the outcome and, if applicable, a summary of the follow-up time. A diagram may be helpful. | 9-10, 15-16 |
|  | 13b | D;V | Describe the characteristics of the participants (basic demographics, clinical features, available predictors), including the number of participants with missing data for predictors and outcome. | 15-16 |
|  | 13c | V | For validation, show a comparison with the development data of the distribution of important variables (demographics, predictors and outcome). | 15-16 |
| Model development | 14a | D | Specify the number of participants and outcome events in each analysis. | 9-10 |
|  | 14b | D | If done, report the unadjusted association between each candidate predictor and outcome. | - |
| Model specification | 15a | D | Present the full prediction model to allow predictions for individuals (i.e., all regression coefficients, and model intercept or baseline survival at a given time point). | 16-19 |
|  | 15b | D | Explain how to the use the prediction model. | 17-19 |
| Model performance | 16 | D;V | Report performance measures (with CIs) for the prediction model. | 16-17 |
| Model-updating | 17 | V | If done, report the results from any model updating (i.e., model specification, model performance). | 16-17 |
| **Discussion** |  |  |  |  |
| Limitations | 18 | D;V | Discuss any limitations of the study (such as nonrepresentative sample, few events per predictor, missing data). | 25-26 |
| Interpretation | 19a | V | For validation, discuss the results with reference to performance in the development data, and any other validation data. | 22 |
|  | 19b | D;V | Give an overall interpretation of the results, considering objectives, limitations, results from similar studies, and other relevant evidence. | 21-26 |
| Implications | 20 | D;V | Discuss the potential clinical use of the model and implications for future research. | 21-26 |
| **Other information** |  |  |  |  |
| Supplementary information | 21 | D;V | Provide information about the availability of supplementary resources, such as study protocol, Web calculator, and data sets. | 30 |
| Funding | 22 | D;V | Give the source of funding and the role of the funders for the present study. | 28-29 |

*Items relevant only to the development of a prediction model are denoted by D, items relating solely to a validation of a prediction model are denoted by V, and items relating to both are denoted D;V

**Supplementary Table 3.** Factors considered for inclusion in the LiFeCRC score.

| **Variable** | **Measurement description in EPIC data** | **Question** |
| --- | --- | --- |
| Age at recruitment, per 10 years | Questionnaire data | How old are you? |
| Waist circumference, per 10 cm | Measurement | Waist circumference was measured either at the narrowest torso circumference or at the midway between the lower ribs and iliac crest. If the narrowest circumference could not be recognised, waist circumference was measured at the midway between the lower ribs and iliac crest. Weight was measured in light underwear in most centers. |
|  | Questionnaire (self-reported)  (in France, the Oxford health conscious, and the Norway cohort. For the study participants from these centers, center specific adjustment formulas were developed and applied) | What is your narrowest waist circumference (without clothing)? |
| Height, per 10 cm | Measurement | Measured with stadiometer. |
|  | Questionnaire (self-reported)  (in France, the Oxford health conscious, and the Norway cohort. For the study participants from these centers, center specific adjustment formulas were developed and applied) | What is your body height? |
| Daily alcohol consumption, yes | Questionnaire data   - Men: > 24 g/day VS ≤ 24 g/day - Women: > 12 g/day VS ≤ 12 g/day | How many standard glasses of beer, wine and distilled spirits did you consume per day or week during the last 12 months? |
| Smoking, yes | Questionnaire data   - current and former smoker VS never smoker | Have you ever been a smoker (*more than one daily* *cigarette, cigar, pipe over a period in lifetime*)? |
| Physically active, yes | Questionnaire data   - active VS inactive | What type of activity was mostly performed during your occupation (*sedentary, standing, manual, heavy manual*) over the last year? For how many hours of recreational (*walking, cycling, sports*) or household activities (*gardening, household work, do-it-yourself work, stairs climbing*) per week did you perform over the last year? Weekly hours are translated into weekly MET-hours and then assigned as *low*, *medium*, *high* or *very* *high*, according to MET-hour quartiles derived from the EPIC study population. *Sedentary* occupation activities paired with *low* to *medium* household and recreational activities were then classified as “inactive”, whereas all other combinations were classified as “active”. |
| Vegetables intake in 100 g/day | Questionnaire data (food frequency questionnaire) | How many grams of vegetables (*leafy vegetables, fruiting vegetables, root vegetables, cabbages, mushrooms, grain and pod vegetables, onion, garlic, stalk vegetables, sprouts, NO soya beans or sprouts*) per day/week/month were you consuming over the last year? |
| Fruits intake in 100 g/day | Questionnaire data (food frequency questionnaire) | How many grams of fruits and fruit compote (*citrus fruits, apple and pear, grape, stone fruits, berries, banana, kiwi, pineapple, date, fig, melon, mango, rhubarb*) per day/week/month were you consuming over the last year? |
| Dark bread intake in 50 g/day | Questionnaire data (food frequency questionnaire) | How many grams of dark bread (*wholemeal bread, rye bread, cereal bread, brown wheat bread)* per day/week/month were you consuming over the last year? |
| Dairy products intake in 100 g/day | Questionnaire data (food frequency questionnaire) | How many grams of dairy products (*milk, milk beverages, yogurt, curd, cheese, cream desserts, milk based puddings, dairy creams, condensed milk*) per day/week/month were you consuming over the last year? |
| Red meat intake in 50 g/day | Questionnaire data (food frequency questionnaire) | How many grams of unprocessed red meat (*beef, veal, pork, mutton/lamb, horse, goat*) per day/week/month were you consuming over the last year? |
| Poultry intake in 50 g/day | Questionnaire data (food frequency questionnaire) | How many grams of unprocessed poultry (*chicken, turkey, duck, goose, domestic rabbit*) per day/week/month were you consuming over the last year? |
| Processed meat intake in 50 g/day | Questionnaire data (food frequency questionnaire) | How many grams of processed meat (*bacon, liver containing items, ham, kassler / cold cuts, hamburger, meatballs, minced meat*) per day/week/month were you consuming over the last year? |
| Fish intake in 50 g/day | Questionnaire data (food frequency questionnaire) | How many grams of fish (*cod, coley, hake, whiting, anchovy, salmon, sardine, tuna*) per day/week/month were you consuming over the last year? |
| Sugar and confectionary intake in 50 g/day | Questionnaire data (food frequency questionnaire) | How many grams of sugar and confectionary (*sugar, honey, jam, syrup, chocolate, sweets, chewing gum, nougat, cereal bar, toffee, peppermint, liquorice, halva, jelly sweets, marzipan, ice cream, sorbet, water ice*) per day/week/month were you consuming over the last year? |
| Soft drinks intake in 100 g/day | Questionnaire data (food frequency questionnaire) | How many grams of soft drinks (*carbonated / soft / isotonic drinks, diluted syrups*) per day/week/month were you consuming over the last year? |

**Supplementary Table 4.** Incidence rates and LifeCRC model selection characteristics across pre-defined risk categories in the derivation and validation samples.

| **Characteristics** | **Derivation cohort** | | |  | **Validation cohort** | | |
| --- | --- | --- | --- | --- | --- | --- | --- |
|  | **Low risk***  <0.62 % | **Intermediate risk***  0.62 to 1.60 % | **High risk*** >1.60 % |  | **Low risk***  0 to 0.62 % | **Intermediate risk***  0.62 to 1.60 % | **High risk*** >1.60 % |
| *N* | 127 741 | 102 192 | 25 549 |  | 37 085 | 303 60 | 6 958 |
| Colorectal cancer incidence, % | 0.6 | 1.9 | 3.6 |  | 0.5 | 1.9 | 3.3 |
| Age at recruitment, years, mean (SD) | 44.3 (7.7) | 57.2 (4.8) | 63.7 (3.6) |  | 42.4 (7.6) | 55.8 (4.5) | 61.8 (3.0) |
| Waist, cm, mean (SD) | 79.8 (11.5) | 87.8 (12.0) | 96.1 (11.8) |  | 80.9 (11.0) | 89.5 (11.4) | 98.6 (10.6) |
| Height, cm, mean (SD) | 164.2 (8.4) | 166.4 (9.4) | 171.0 (9.0) |  | 165.9 (9.6) | 168.0 (9.5) | 172.4 (8.3) |
| Daily alcohol consumption, % high | 22.5 | 31.9 | 38.1 |  | 22.9 | 32.4 | 43.9 |
| Ever smoker, % yes | 42.9 | 54.3 | 76.6 |  | 48.4 | 56.1 | 82.2 |
| Physically active, % yes | 81.5 | 79.7 | 82.2 |  | 83.0 | 79.9 | 82.8 |
| Dietary intake, g/day, median (IQR) |  |  |  |  |  |  |  |
| Vegetables | 232.7  (149.1 - 345.2) | 173.1  (112.3 - 263.2) | 142.3  (92.3 - 218.2) |  | 130.1  (94.5 - 184.7) | 133.0  (93.2 - 186.4) | 117.0  (82.4 - 160.8) |
| Dairy products | 284.1  (166.6 - 435.6) | 281.9  (153.6 - 458.0) | 283.8  (145.1 - 464.1) |  | 272.9  (159.5 - 440.9) | 264.3  (145.2 - 438.9) | 232.0  (121.3 - 380.7) |
| Processed meat | 15.2  (3.6 - 32.7) | 22.0  (9.9 - 40.6) | 29.4  (13.9 - 52.1) |  | 32.0  (16.1 - 56.5) | 34.9  (18.9 - 59.3) | 48.6  (27.6 - 77.0) |
| Sugar and confectionary | 28.4  (14.9 - 49.2) | 34.3  (17.7 - 59.7) | 40.5  (20.9 - 71.4) |  | 33.2  (17.3 - 56.9) | 37.3  (20.0 - 62.9) | 38.0  (21.1 - 67.2) |
| *Risk groups are based on the 50^th^ and 90^th^ percentile of predicted 10-year absolute risks of colorectal cancer using the LiFeCRC score, i.e. participants are categorized as *low risk* when below the 50^th^ percentile (0.62 %), *high risk* when above the 90^th^ percentile (1.60 %), and *intermediate risk* when between the 50^th^ and 90^th^ percentiles.  SD – standard deviation; IQR – interquartile range | | | | | | | |

**Supplementary Table 5.** Added predictive performance for age and lifestyle-based (LiFeCRC) colorectal cancer risk prediction models. Results are stratified by age groups.

| **Statistics** | **Derivation data** | | | |  |
| --- | --- | --- | --- | --- | --- |
|  | **All ages**  (*N* = 255 482) | **< 45 years** (*N* = 60 099) | **45 - 65** **years** (*N* = 143 692) | **> 65** **years** (*N* = 51 691) |  |
| **Harrell‘s C-index**  (95% confidence interval)* |  |  |  |  |  |
| Age | 0.690  (0.682 to 0.698) | 0.639  (0.605 to 0.673) | 0.603  (0.591 to 0.616) | 0.518  (0.503 to 0.534) |  |
| LiFeCRC | 0.710  (0.702 to 0.718) | 0.662  (0.627 to 0.698) | 0.636  (0.624 to 0.649) | 0.591  (0.576 to 0.606) |  |
| Δ LiFeCRC / Age | 0.019  (0.019 to 0.020) | 0.023  (0.022 to 0.025) | 0.033  (0.033 to 0.034) | 0.073  (0.072 to 0.074) |  |
| *P*_difference_ LiFeCRC / Age | < 0.001 | < 0.001 | < 0.001 | < 0.001 |  |
|  |  |  |  |  |  |
| **Likelihood ratio test**† |  |  |  |  |  |
| *P*_LRT_ Age / LiFeCRC | < 0.001 | 0.023 | < 0.001 | < 0.001 |  |
|  |  |  |  |  |  |
| **NRI^> 0^**  (95% confidence interval)‡ |  |  |  |  |  |
| Age / LiFeCRC | 0.307  (0.264 to 0.352) | 0.364  (0.084 to 0.575) | 0.263  (0.205 to 0.326) | 0.304  (0.217 to 0.366) |  |
| *Harrell’s C-index with 95% confidence interval based on 1000-fold bootstrapping. Harrell’s C-index difference Δ of the LiFeCRC model and a model based on age only. *P*_difference_ of a paired t-test for significant differences in Harrell’s C-index of the LiFeCRC model and a model based on age only.  †Test for significant improvement in goodness of fit between a base model (age) and an extended model (LiFeCRC).  ‡Continuous NRI (net reclassification improvement) index, quantifies the improvement in category-free risk reclassification between a base model (age) and an extended model (LiFeCRC). | | | | | |

**Supplementary Table 6.** Sensitivity analysis in subgroups for the colorectal cancer model for both sexes.

| **Stratification** | **Harrell’s C-index (95% confidence interval)** | | | | | |
| --- | --- | --- | --- | --- | --- | --- |
|  | **Derivation cohort** | | | **Validation cohort** | | |
|  | ***N*** | **Full model** | **Reduced model  (LiFeCRC score)** | ***N*** | **Full model** | **Reduced model  (LiFeCRC score)** |
| Full derivation sample | 255 482 | 0.710 (0.702 to 0.718) | 0.710 (0.702 to 0.718) | 74 403 | 0.715 (0.699 to 0.730) | 0.714 (0.699 to 0.730) |
|  |  |  |  |  |  |  |
| Waist circumference* |  |  |  |  |  |  |
| Low | 158 872 | 0.716 (0.705 to 0.727) | 0.716 (0.705 to 0.727) | 44 180 | 0.715 (0.692 to 0.734) | 0.713 (0.690 to 0.736) |
| High | 96 610 | 0.683 (0.671 to 0.695) | 0.682 (0.670 to 0.695) | 30 223 | 0.680 (0.657 to 0.703) | 0.680 (0.658 to 0.702) |
|  |  |  |  |  |  |  |
| Education† |  |  |  |  |  |  |
| Lower | 192 606 | 0.699 (0.690 to 0.709) | 0.699 (0.691 to 0.708) | 57 298 | 0.706 (0.689 to 0.723) | 0.705 (0.687 to 0.722) |
| University degree | 62 876 | 0.737 (0.719 to 0.754) | 0.736 (0.718 to 0.752) | 17 105 | 0.750 (0.714 to 0.784) | 0.751 (0.716 to 0.785) |
|  |  |  |  |  |  |  |
| Smoking |  |  |  |  |  |  |
| Never | 125 523 | 0.706 (0.694 to 0.718) | 0.706 (0.694 to 0.718) | 33 680 | 0.701 (0.675 to 0.729) | 0.701 (0.676 to 0.728) |
| Current or former | 129 959 | 0.705 (0.694 to 0.715) | 0.704 (0.694 to 0.715) | 40 723 | 0.716 (0.697 to 0.736) | 0.715 (0.696 to 0.734) |
| Current, 1-15 cig/day | 29 225 | 0.694 (0.669 to 0.718) | 0.693 (0.669 to 0.718) | 10 537 | 0.722 (0.678 to 0.764) | 0.721 (0.679 to 0.762) |
| Current, 16+ cig/day | 22 688 | 0.722 (0.694 to 0.749) | 0.721 (0.694 to 0.749) | 7 013 | 0.708 (0.664 to 0.751) | 0.704 (0.659 to 0.747) |
| Daily alcohol consumption‡ |  |  |  |  |  |  |
| Low | 184 393 | 0.715 (0.706 to 0.725) | 0.715 (0.706 to 0.724) | 53 035 | 0.717 (0.698 to 0.735) | 0.716 (0.696 to 0.735) |
| High | 71 089 | 0.693 (0.677 to 0.707) | 0.692 (0.677 to 0.706) | 21 368 | 0.699 (0.675 to 0.724) | 0.699 (0.674 to 0.724) |

*Waist circumference was divided into “low” and “high” using a cut point of 94 cm for men and 84 cm for women.

†Education was divided into “university degree” and all others as “lower”.

‡Daily alcohol consumption was divided into “low” and “high” using a cut point of 24 g/day for men and 12g/day for women.
