# Supplementary material for: Development and validation of a lifestyle-based model for colorectal cancer risk prediction: the LiFeCRC score
Source: BMC Med. 2021 Jan 4;19:1. doi: 10.1186/s12916-020-01826-0 (PMC7780676; doi:10.1186/s12916-020-01826-0)
Supplement: Supplementary file 2 — Additional file 2: Supplementary Figure 1. Workflow of risk model development and validation. Supplementary Figure 2. Survival function of average predictor values of the derivation cohort. Supplementary Figure 3. Discrimination and relative variable importance based on Random Survival Forest models for colorectal cancer prediction. Supplementary Figure 4. Random Survival Forest colorectal cancer full model calibration. Supplementary Figure 5. Model performance comparison the LiFeCRC score and a colorectal cancer risk model including only age. (a) Calibration plot of predicted 10-year colorectal cancer risk for a model that included only age and the LiFeCRC score model with additional lifestyle predictors (waist circumference, body height, daily alcohol consumption, smoking, physical activity, and daily intake of vegetables, dairy products and red meat). (b) Decision curves illustrating net benefit of prediction models for a range of colorectal cancer risk thresholds, used to decide about further treatment or intervention. Decisions curves are shown for different models: none treatment, all treatment, treatment based on the age-model, treatment based on the LiFeCRC model. Supplementary Figure 6. Predicted 10-year absolute risk of colorectal cancer for a healthy and unhealthy lifestyle. Risk across different age-groups and a constant body height of 166 cm. Unhealthy lifestyle: waist circumference of 100 cm, high daily alcohol consumption, smoker, physically inactive, 80 g daily vegetable intake, 70 g daily dairy products intake, 60 g daily processed meat intake, and 90 g daily sugar and confectionary intake. Healthy lifestyle: waist circumference of 70 cm, low daily alcohol consumption, non-smoker, physically active, 430 g daily vegetable intake, 630 g daily dairy products intake, 0 g daily processed meat intake, and 5 g daily sugar and confectionary intake. Supplementary Figure 7. Full model performance including NSAID use and family history of colorectal cancer. [file 12916_2020_1826_MOESM2_ESM.docx]

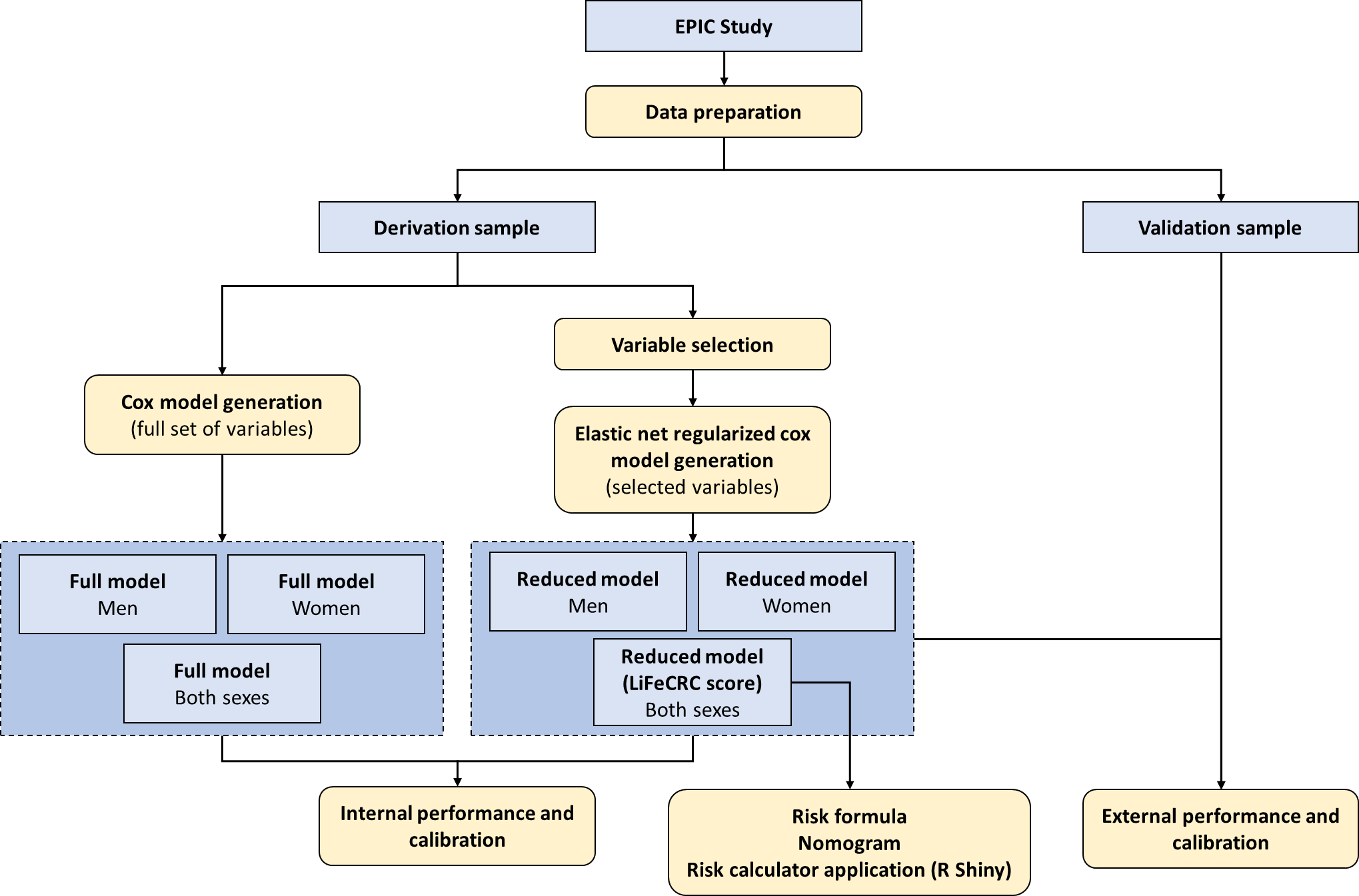


**Supplementary Figure 1.** Workflow of risk model development and validation.


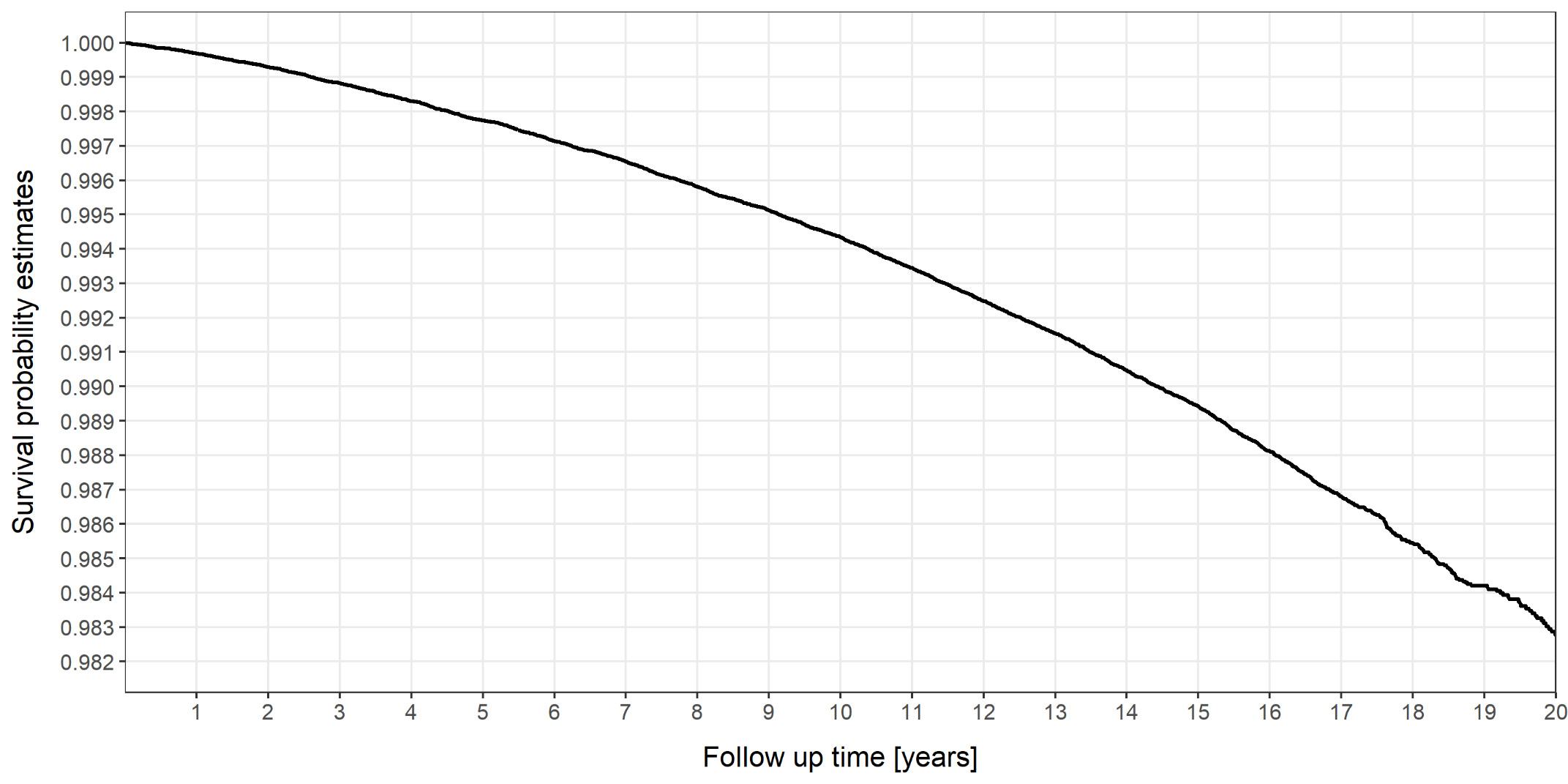


**Supplementary Figure 2.** Survival function of average predictor values of the derivation cohort.


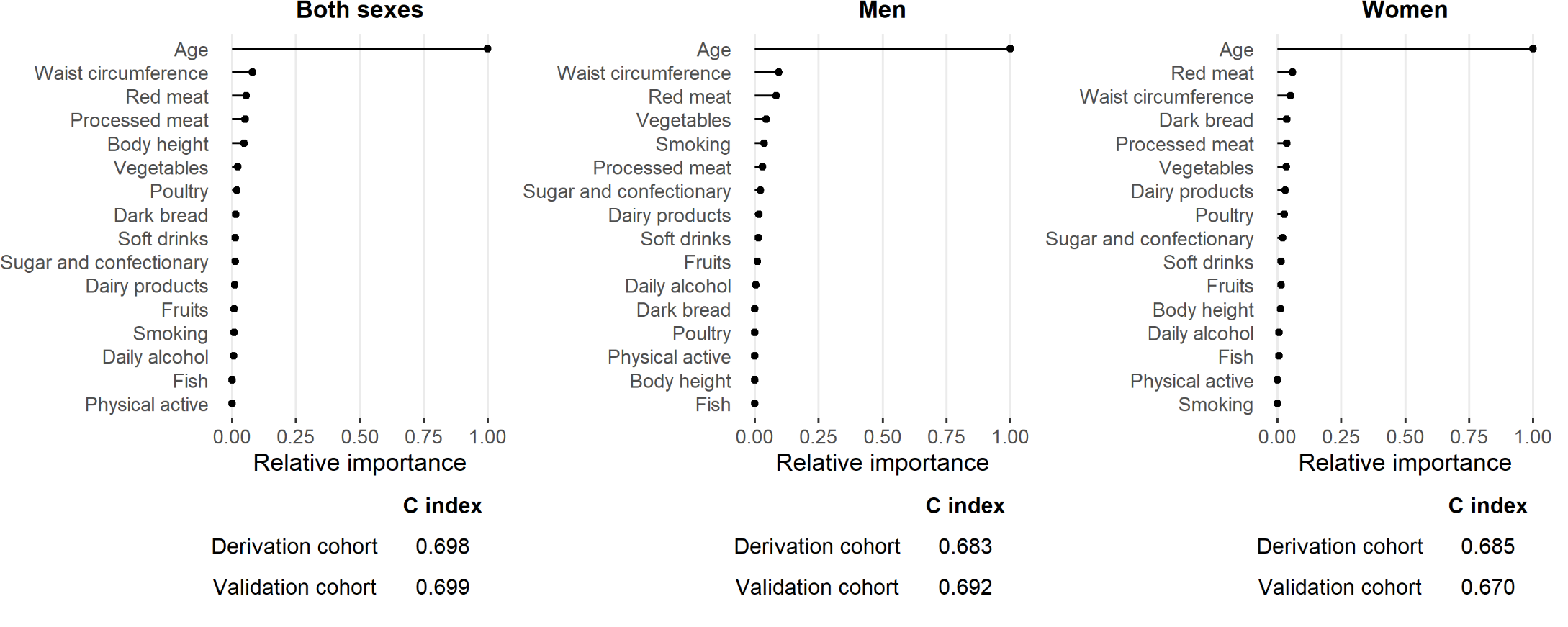


**Supplementary Figure 3.** Discrimination and relative variable importance based on Random Survival Forest models for colorectal cancer prediction.


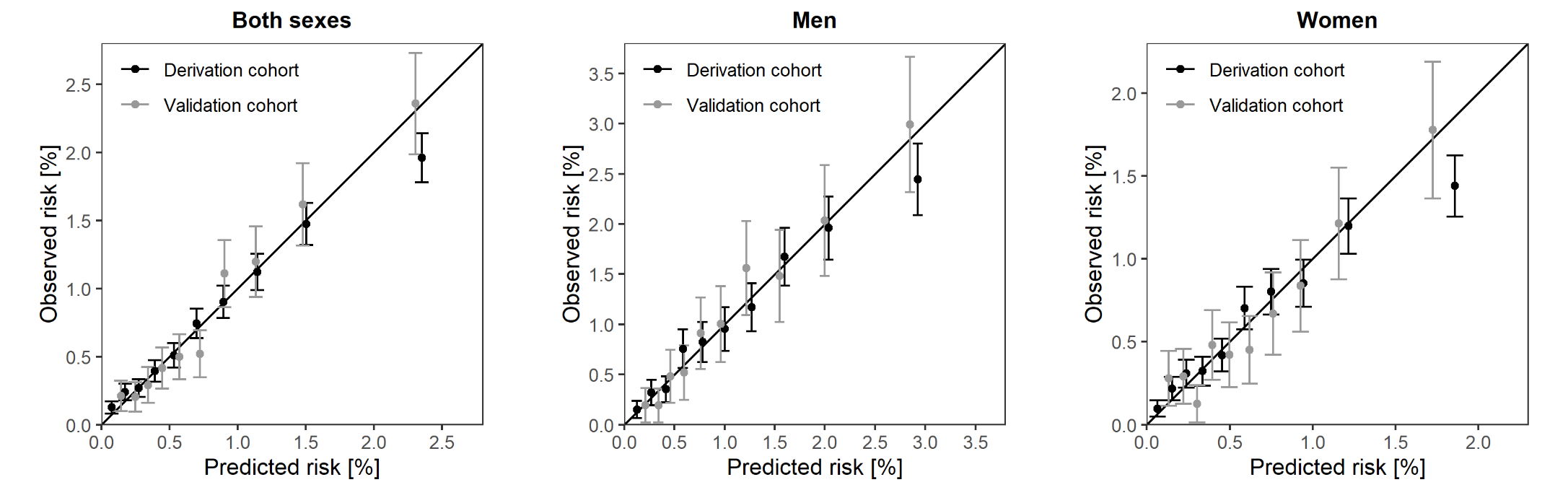


**Supplementary Figure 4.** Random Survival Forest colorectal cancer full model calibration.


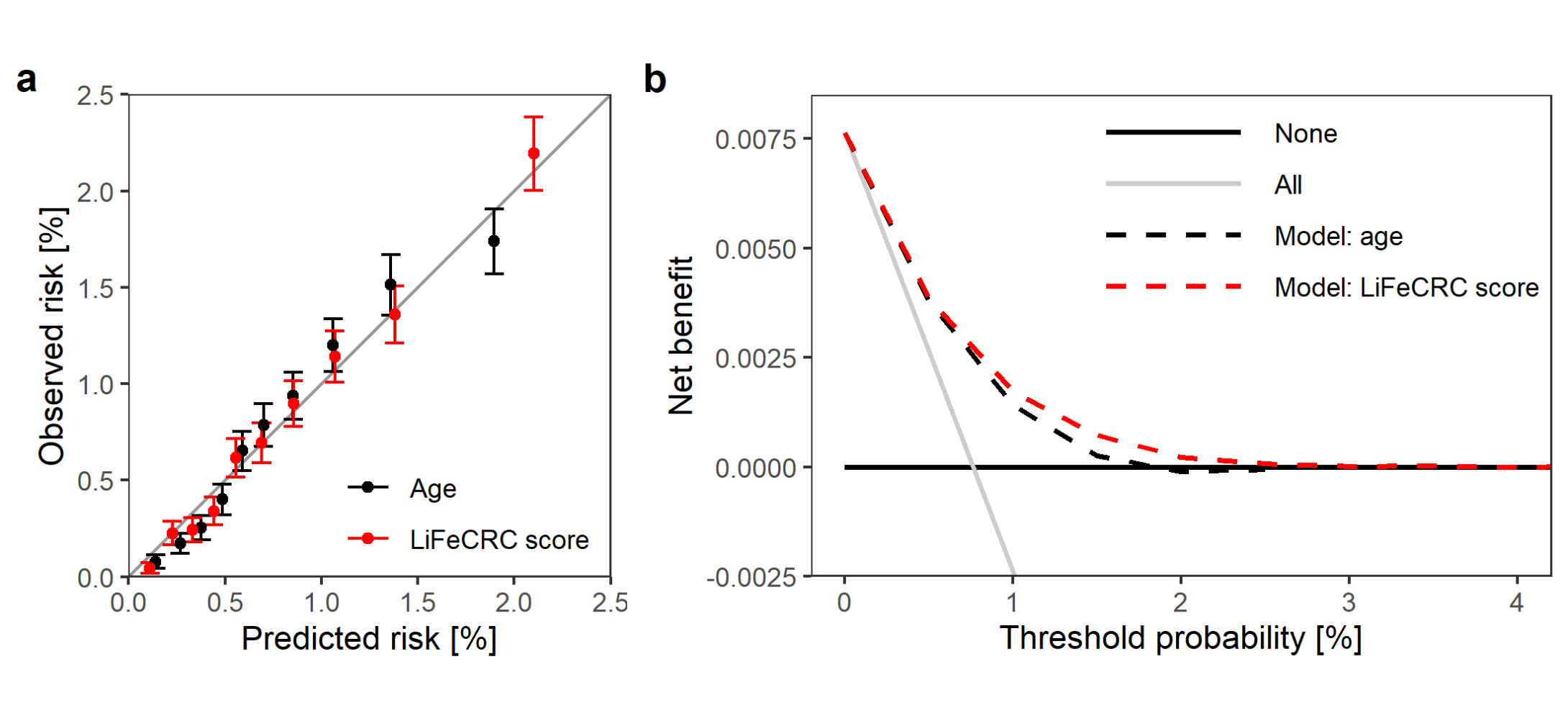


**Supplementary Figure 5.** Model performance comparison of the LiFeCRC score and a colorectal cancer risk model including only age.
(a) Calibration plot of predicted 10-year colorectal cancer risk for a model that included only age and the LiFeCRC score model with additional lifestyle predictors (waist circumference, body height, daily alcohol consumption, smoking, physical activity, and daily intake of vegetables, dairy products and red meat). (b) Decision curves illustrating net benefit of prediction models for a range of colorectal cancer risk thresholds, used to decide about further treatment or intervention. Decisions curves are shown for different models: none treatment, all treatment, treatment based on the age-model, treatment based on the LiFeCRC model.


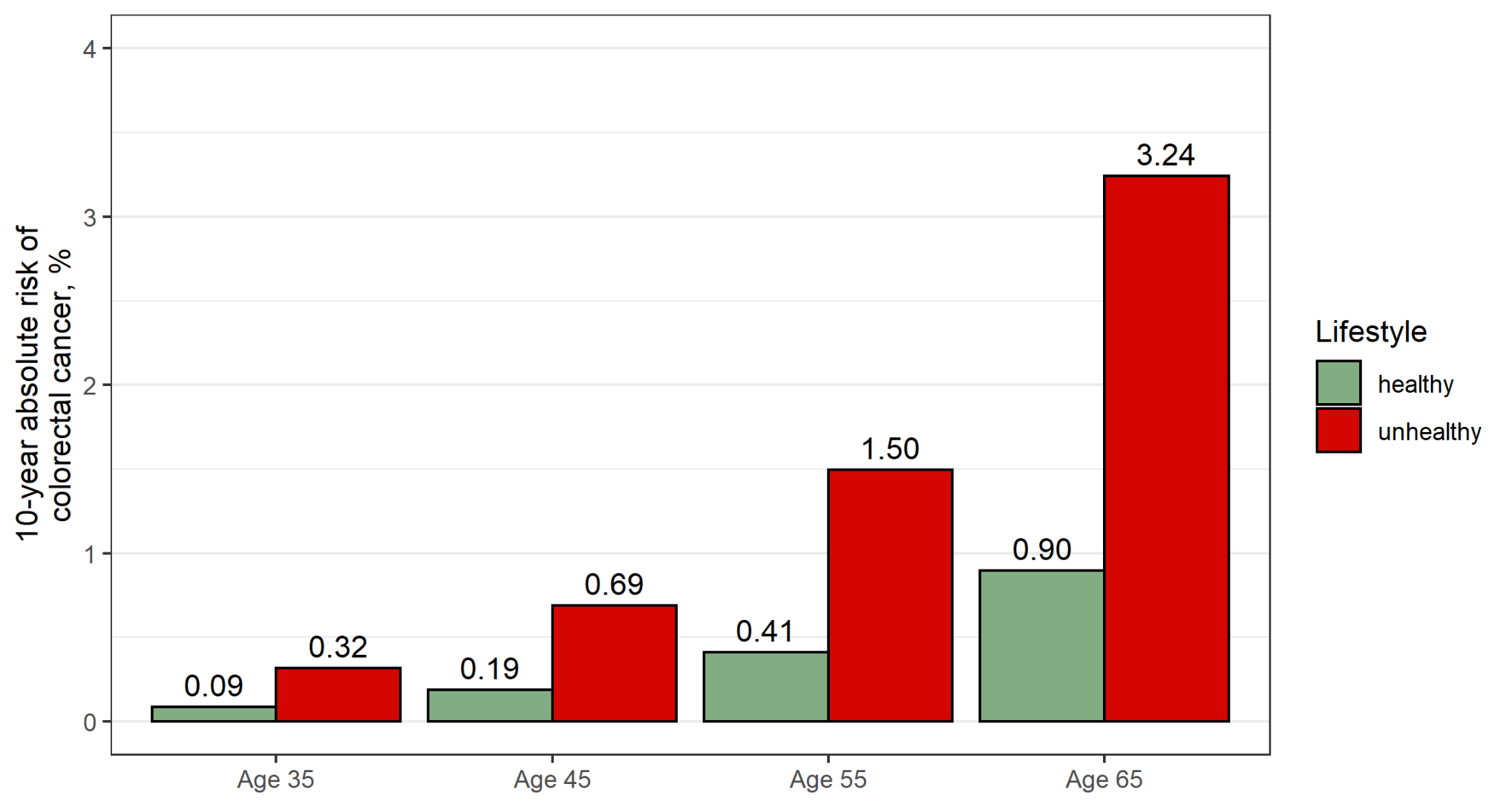


**Supplementary Figure 6.** Predicted 10-year absolute risk of colorectal cancer for a healthy and unhealthy lifestyle. Risk across different age-groups and a constant body height of 166 cm. *Unhealthy lifestyle:* waist circumference of 100 cm, high daily alcohol consumption, smoker, physically inactive, 80 g daily vegetable intake, 70 g daily dairy products intake, 60 g daily processed meat intake, and 90 g daily sugar and confectionary intake. *Healthy lifestyle:* waist circumference of 70 cm, low daily alcohol consumption, non-smoker, physically active, 430 g daily vegetable intake, 630 g daily dairy products intake, 0 g daily processed meat intake, and 5 g daily sugar and confectionary intake.


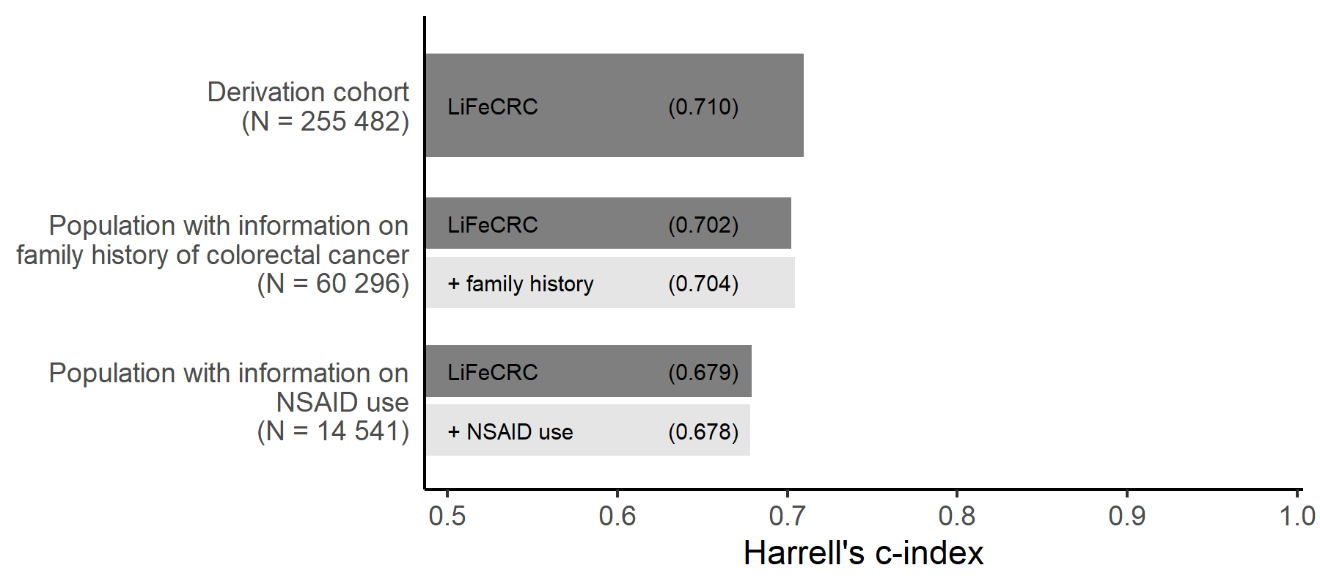


**Supplementary Figure 7.** Full model performance including NSAID use and family history of colorectal cancer.
